# Supplementary material for: Eye size and investment in frogs and toads correlate with adult habitat, activity pattern and breeding ecology
Source: Proc Biol Sci. 2020 Sep 23;287(1935):20201393. doi: 10.1098/rspb.2020.1393 (PMC7542830; doi:10.1098/rspb.2020.1393)
Supplement: Supplementary Materials [file rspb20201393supp1.pdf]

Electronic Supplementary Materials for:

Eye size and investment in frogs and toads correlate with adult habitat, activity pattern, and breeding ecology

Kate N. Thomas, David J. Gower, Rayna C. Bell, Matthew K. Fujita, Ryan K. Schott, Jeffrey W. Streicher

Proceedings of the Royal Society B: Biological Sciences

DOI: 10.1098/rspb.2020.1393

Supporting Information:

Supplemental Methods

Supplemental Results

Tables S1-S10

Figures S1-S11

Supplemental References

Supplemental Methods

Validating specimen measurements (expanded)

Fresh specimens (n = 67) were euthanized and then immediately measured for all measurements outlined previously for museum specimens. After measuring transverse eye diameters externally, one eye was carefully dissected whole out of the specimen and transverse eye diameter measured again. Both eyes were subsequently used for other research, so remeasuring the same specimens after preservation was not possible.

We first used OLS regression to determine how externally measured ED correlates with dissected ED in fresh specimens. We then used SMA tests in *smatr* to determine whether preserved (museum) and fresh specimens exhibit significantly different eye-body allometric relationships, both for externally measured ED in both datasets, and for externally measured ED in preserved specimens vs. dissected. whole eye measurements in fresh specimens.

Comparison to a previous study

A recent study by Huang et al. (2019) of 44 anurans from eight families produced a dataset including SVL, mass, and CD (we back-transformed published eye volume approximations to raw CD measurements using an equation provided in that publication). We used SMA tests in *smatr* to compare allometric relationships between 1) CD and RM and 2) CD and SVL in that study and the present study.

This recent study also found no correlations between relative eye size (approximated from cornea diameters) and habitat type (terrestrial/arboreal vs. aquatic/semiaquatic) or activity pattern (nocturnal vs. cathemeral). Our finding of significant effects of both activity

pattern and habitat on relative eye size may result from our increased sampling and inclusion of broad ecological and taxonomic diversity. However, we also categorized adult habitat into 6 states (semiaquatic, aquatic, scansorial, ground-dwelling, subfossorial, fossorial), while the previous study used binary states (aquatic/semiaquatic vs. terrestrial/arboreal). To test whether this difference contributed to our new findings, we recategorized our species data into the binary habitat states found in Huang et al. (2019) by putting aquatic and semiaquatic species into the "aquatic/semiaquatic" category, and scansorial, ground-dwelling, fossorial, and subfossorial species into the "terrestrial/arboreal" category. We then used Kruskal-Wallis tests to test whether EDs, eye investment relative to RM, or eye investment relative to SVL differed among recategorized binary habitat states.

Supplemental Results

Museum specimen measurements are reliable for eye-body allometry (expanded)

In fresh specimens, ED measured from whole dissected eyes was highly correlated with ED measured externally prior to dissection ($R^2 = 0.96$, n = 55, $SE_{res.} = 0.04$, $F_{(1,53)} = 1387$, $p < 0.0001$). External ED predicted dissected ED in a log-transformed OLS regression with an allometric slope of 1.01 (SE = 0.03, t = 37.2, p < 0.0001) and intercept of -0.02 (SE = 0.14, t = -1.11, p = 0.27), indicating that external measurement slightly overestimates eye size but is a reasonable and consistent approximation (Figure S2B). AL measured from whole dissected eyes was also highly correlated with ED measured externally prior to dissection ($R^2 = 0.96$, n = 52, $SE_{res.} = 0.04$, $F_{(1,50)} = 1276$, $p < 0.0001$). ED predicted AL in a log-transformed OLS regression with an allometric slope of 1.02 (SE = 0.03, t = 35.7, p < 0.0001) and intercept of –0.06 (SE = 0.02, t =

-2.64, p = 0.01, Figure S2A). While there is some variation in this relationship, the high correlation indicates that it is reasonable to use external transverse ED to approximate AL among anurans.

Datasets collected from fresh (externally measured and dissected ED) and preserved (externally measured ED) specimens yielded similar allometric regressions for eye scaling with body size. Allometric relationships between external ED and RM did not significantly differ in slopes (SMA test: Likelihood ratio stat. = 2.08, df = 1, p = 0.15) or intercepts (Wald stat. = 0.33, df = 1, p = 0.56) between fresh and preserved specimens (Figure S2C). Further, eye-body mass allometry derived using whole, dissected ED in fresh specimens did not show significant differences in slope (SMA test: Likelihood ratio stat. = 0.43, df = 1, p = 0.51) or intercept (Wald stat. = 2.16, df = 1, p = 0.14) from the relationship derived from externally measured ED in preserved specimens, indicating that the combination of external eye measurement and preservation introduce negligible error to estimates of anuran eye-body mass allometry (Figure S2E).

By contrast, the allometric scaling of external ED with SVL had significantly different slopes in fresh and preserved specimens (SMA test: Likelihood ratio stat. = 5.31, df = 1, p = 0.02), with a higher slope in preserved (0.94) than fresh specimens (0.83). Additionally, eye-SVL allometry derived using whole, dissected ED in fresh specimens did not show a significant difference in slope (SMA test: Likelihood ratio stat. = 2.40, df = 1, p = 0.12) from the relationship derived from externally measured ED in preserved specimens, but did show a significant difference in intercept (Wald stat. = 7.18, df = 1, p = 0.007), with a slightly higher intercept in preserved (-0.79) than in fresh specimens (-0.81, Figure S2F). Together, these results indicate

that that ED:SVL in preserved specimens may yield slight overestimates of relative eye size compared to ED:RM, perhaps due to shrinkage in preserved specimens. However, these differences may also result from differences in species sampling across preserved and fresh specimen datasets. Regardless of cause, the differences in allometric estimates are small enough in magnitude that SVL appears to be a reasonable measure for anuran eye-body allometry if mass data are not available.

Eye-body allometry is consistent with a previous study

SMA tests comparing the scaling of CD with RM in our museum specimen data with data published by Huang et al. (2019) showed no difference in SMA slopes (Likelihood ratio stat.: 2.52, df = 3, $p = 0.47$) but a difference in intercepts (Wald stat.: 46.8, df = 3, $p < 0.0001$); the same pattern was observed for SVL (Figure S10). In both comparisons, the previously published data showed a slightly lower intercept; however, this was not a result of preservation or sampling bias at the family level, because SMA lines fitted to our own data from fresh specimens and to a subset of our museum data including only the families sampled in the photograph-based data set both yielded fits not significantly different from our full museum specimen dataset (Figure S10). Thus we conclude that, (1) the difference in intercepts is best explained by differences in measurement techniques (direct measurement vs. photographic measurement) and/or sample sizes and (2) our measurement protocol produced comparable results whether applied to museum or fresh specimens.

Habitat effects are masked in binary states

Recategorization of our species data into the binary habitat states used by Huang et al. (2019) replicated their finding that relative eye size does not differ across habitats (Figure S11). Absolute eye diameter significantly differed across binary habitat states (Kruskal-Wallis: χ^2 = 8.47, df = 1, p = 0.004). However, relative eye investment did not differ across states whether measured against RM (Kruskal-Wallis: χ^2 = 0.03, df = 1, p = 0.87) or SVL (Kruskal-Wallis: χ^2 = 0.91, df = 1, p = 0.34).

Supplemental Figures and Tables

Table S1. Criteria used to assign species to states for ecological traits used in analyses.

Trait states	Definition
**Adult habitat**	
Semiaquatic	Strongly associated with/commonly found in water but also frequently uses land habitats
Aquatic	Primarily found in water; rarely/never leaves water
Scansorial	Primarily associated with plants; up off the ground (arboreal/shrubs/reeds)
Ground-dwelling	Primarily active on the ground
Subfossorial	Primarily active under leaf litter; shallow burrowers
Fossorial	Primarily active in deeper burrows (not burrowing simply for aestivation/long periods of inactivity)
**Activity period**	
Diurnal	Primarily active in daylight above ground
Nocturnal	Primarily active at night above ground
Both	Regularly active during both day and at night or exclusively at transition (crepuscular)
**Mating habitat**	
Lotic water	In or near moving (lotic) water (e.g. streams, rivers)
Lentic water	In or near still (lentic) water or very slow-moving water (e.g. ponds, puddles)
Plants	On/associated with plants
Ground	On ground or leaf litter
**Life history**	
Free-living larvae	Biphasic with a free-living larval stage (i.e. free-living tadpoles)
No free-living larvae	No free-living tadpoles (direct development, viviparity, marsupiality)
**Larval habitat**	
No larvae	No free-living larvae
Lotic water	Primarily active in flowing water (e.g. streams, rivers)
Lentic water	Primarily active in still water (e.g. puddles, ponds, water trapped in plants)
On land	Primarily active on the ground or in leaf litter
Obscured	Primarily active within sand/mud/soil, foam nests in chambers, or caves
**Sexual dichromatism**	
Present	Breeding females and males are markedly different colors/patterns (either developmental or dynamic)
Absent	Breeding females and males are similar colors/patterns

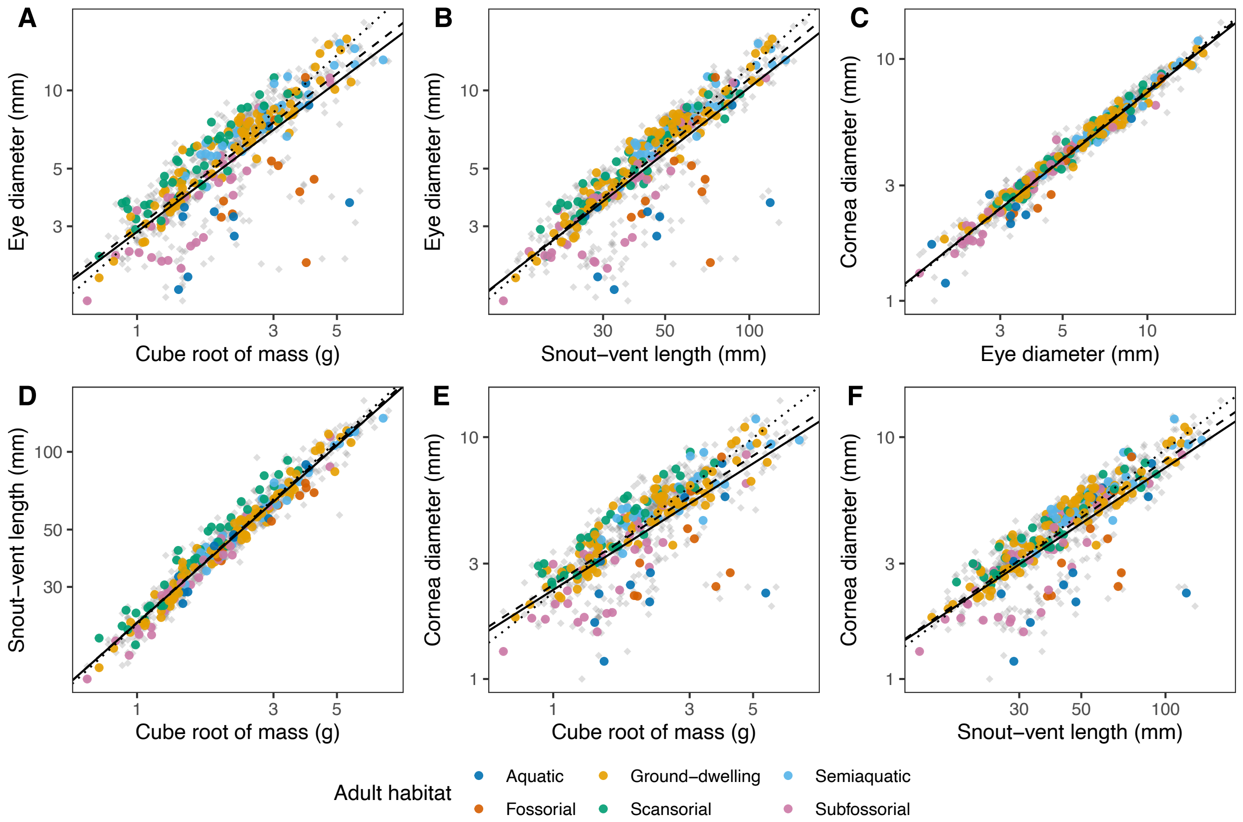

Figure S1. Allometric scaling relationships between measures of eye size and body size across anurans. Specimen measurements (n = 640) are plotted in gray diamonds, and species means (n = 220, used for regressions) in circles colored by adult habitat. Scaling of eye diameter with **(A)** the cube root of body mass and **(B)** snout-vent length (SVL) were similar to each other and to the scaling of corneal diameter with **(E)** the cube root of mass and **(D)** SVL. This is because cornea diameter was highly correlated with eye diameter **(C)**, and SVL with the cube root of mass **(D)**. Phylogenetic generalised least squares regressions (solid) of eye and cornea scaling with body size (A, B, C, D) were consistently lower in slope than either ordinary least squares regressions (dashed) or standardised major axis regressions (dotted).

Table S2. Parameter estimates for phylogenetic generalised least squares (PGLS) models of allometric relationships between measures of anuran eye size and body size. Models used logged species means of morphological measurements. ED = mean eye diameter, RM = cube root of mass, SVL = snout-vent length, CD = corneal diameter.

**Parameter**	**PGLS model**					
	**ED vs. RM**	**ED vs. SVL**	**CD vs. ED**	**SVL vs. RM**	**CD vs. RM**	**CD vs. SVL**
λ	0.96	0.96	0.40	0.75	0.88	0.87
95% conf.	0.90, 0.99	0.90, 1.00	0.06, 0.65	0.53, 0.87	0.78, 0.94	0.78, 0.94
Residual SE	0.11	0.10	0.03	0.04	0.10	0.09
Adjusted R^2	0.80	0.84	0.96	0.96	0.73	0.78
F-statistic	861.9	1177	5266	4656	577.3	790.6
df	1, 213	1, 218	1, 216	1, 213	1, 211	1, 216
p-value	< 0.0001	< 0.0001	< 0.0001	< 0.0001	< 0.0001	< 0.0001
Slope	0.82	0.84	0.92	0.98	0.74	0.76
SE	0.03	0.02	0.01	0.01	0.03	0.03
t-value	29.4	34.3	72.6	68.2	24.0	28.1
p-value	< 0.0001	<0.0001	<0.0001	< 0.0001	< 0.0001	< 0.0001
Intercept	0.46	-0.66	-0.06	1.34	0.37	-0.66
SE	0.06	0.07	0.01	0.02	0.06	0.07
t-value	7.29	-9.72	-3.85	62.7	6.64	-9.89
p-value	< 0.0001	<0.0001	0.0002	< 0.0001	< 0.0001	< 0.0001

Table S3. Iterative removal of potential phylogenetic outliers (species with phylogenetic studentized residuals > |±3|) from phylogenetic generalised least squares (PGLS) models has little to no impact on parameter estimates for the allometric scaling relationships examined. Models were run iteratively until no phylogenetic outliers remained or 10 iterations were complete. ED = mean eye diameter, RM = cube root of mass, SVL = snout-vent length, CD = mean cornea diameter.

**Model/ iteration**	**Phylogenetic outlier species removed (cumulative)**	**N**	**Slope**	**Intercept**	**λ (95% conf.)**
ED vs. RM					
1	none	215	0.82	0.46	0.96 (0.90, 0.99)
2	*Megophrys gerti, Oreobtes quixensis*	213	0.82	0.46	0.96 (0.90, 0.99)
3	*Megophrys microstoma, Strabomantis bufoniformis*	211	0.82	0.46	0.96 (0.90, 0.99)
4	*Megophrys brachykolos, Thoropa miliaris*	209	0.82	0.46	0.96 (0.90, 0.99)
5	*Leptobrachella dringi, Proceratophrys boiei*	207	0.82	0.46	0.96 (0.90, 0.99)
6	*Kalophrynus interlineatus*	206	0.82	0.46	0.96 (0.90, 1.00)
7	*Rentapia hosii*	205	0.82	0.46	0.96 (0.90, 1.00)
8	*Phrynoidis juxtaspera*	204	0.81	0.46	0.97 (0.91, NA)
9	*Gastrophrynoides borneensis, Callulina kreffti*	202	0.81	0.46	0.97 (0.92, NA)
10	*Kalophrynus pleurostigma, Rana temporaria*	200	0.81	0.46	0.97 (0.92, NA)
ED vs. SVL					
1	none	220	0.84	-0.66	0.96 (0.90, 1.00)
2	*Heleophryne purcelli, Calyptocephalella gayi*	218	0.84	-0.67	0.96 (0.90, 1.00)
3	*Leptobrachium chapaense, Mixophyes fasciolatus*	216	0.84	-0.67	0.96 (0.90, 1.00)
4	*Limnodynastes salmini*	215	0.84	-0.67	0.96 (0.90, 1.00)
5	*Batrachyla leptopus, Aglyptodactylus madagascariensis*	213	0.84	-0.67	0.96 (0.90, 1.00)
6	*Batrachyla taeniata*	212	0.84	-0.67	0.96 (0.90, 1.00)
7	*Telmatobius culeus*	211	0.84	-0.67	0.96 (0.90, 1.00)
8	*Lepidobatrachus laevis, Microhyla fusca*	209	0.84	-0.67	0.96 (0.90, 1.00)
9	*Hylodes nasus, Microhyla pulverata*	207	0.84	-0.67	0.96 (0.90, 1.00)
10	*Gastrophrynoides borneensis*	206	0.83	-0.66	0.96 (0.90, 1.00)
ED vs. CD					
1	none	218	0.92	-0.06	0.40 (0.06, 0.65)
2	*Spelaeophryne methneri*	217	0.92	-0.06	0.40 (0.07, 0.66)
3	*Breviceps mossambicus, Aglyptodactylus madagascariensis*	215	0.92	-0.06	0.39 (0.06, 0.65)
4	*Bombina orientalis, Aplastodiscus albofrenatus*	213	0.92	-0.06	0.40 (0.07, 0.65)
5	*Ptychadena oxyrhynchus*	212	0.92	-0.06	0.40 (0.07, 0.65)
6	*Bombina fortinuptialis, Conraua crassipes*	210	0.92	-0.06	0.41 (0.08, 0.66)
7	*Rhinophrynus dorsalis*	209	0.93	-0.06	0.00 (NA, 0.63)
SVL vs. RM					
1	none	215	0.98	1.34	0.75 (0.53, 0.87)
2	*Rana pipiens*	214	0.98	1.34	0.75 (0.54, 0.87)
3	*Pipa pipa*	213	0.98	1.34	0.74 (0.53, 0.87)
CD vs. RM					
1	none	213	0.75	0.37	0.88 (0.78, 0.94)
2	*Aglyptodactylus madagascariensis*	212	0.75	0.37	0.88 (0.78, 0.94)
3	*Bombina orientalis, Megophrys gerti, Breviceps mossambicus*	209	0.75	0.37	0.88 (0.77, 0.94)
4	*Alytes obstetricans, Micrixalus phyllophilus*	207	0.75	0.36	0.88 (0.78, 0.94)
5	*Discoglossus pictus, Pipa carvalhoi*	205	0.77	0.34	0.94 (0.87, 0.98)
6	*Leiopelma hochstetteri*	204	0.77	0.32	0.94 (0.86, 0.98)
7	*Leptodactylus melanonotus*	203	0.76	0.32	0.94 (0.86, 0.98)

8	*Barbourula busuangensis, Incilius nebulifer*	201	0.78	0.30	0.96 (0.90, 0.99)
9	*Leptodactylus bolivianus*	200	0.79	0.30	0.96 (0.89 0.99)
CD vs. SVL					
1	none	218	0.76	-0.66	0.87 (0.78, 0.94)
2	*Alytes obstetricans, Bombina orientalis, Allophryne ruthveni, Aglyptodactylus madagascariensis*	214	0.76	-0.66	0.87 (0.77, 0.94)
3	*Discoglossus pictus, Rhinophrynus dorsalis, Mantella aurantiaca*	211	0.76	-0.65	0.86 (0.76, 0.93)
4	*Leiopelma hochstetteri, Eleutherodactylus planirostris*	209	0.76	-0.67	0.86 (0.76, 0.93)
5	*Hyla cinerea*	208	0.77	-0.67	0.86 (0.76, 0.94)
6	*Eleutherodactylus coqui*	207	0.77	-0.67	0.87 (0.76, 0.94)
7	*Craugastor fitzingeri*	206	0.77	-0.67	0.87 (0.76, 0.94)
8	*Diasporus diastema*	205	0.77	-0.67	0.87 (0.76, 0.94)
9	*Craugstor podiciferus, Tricobatrachus robustus*	203	0.77	-0.68	0.87 (0.76, 0.94)
10	*Bombina fortinuptialis, Eleutherodactylus marnockii*	201	0.77	-0.68	0.87 (0.76, 0.94)

Table S4. Parameter estimates for ordinary least squares (OLS) and standardized major axis (SMA) models fit to the same logged species data for anuran eye and body size used in phylogenetic generalised least squares (PGLS) models. ED = mean eye diameter, RM = cube root of mass, SVL = snout-vent length, CD = mean cornea diameter.

	**Comparison**					
**Model and outputs**	**ED vs. RM**	**ED vs. SVL**	**CD vs. ED**	**SVL vs. RM**	**CD vs. RM**	**CD vs. SVL**
OLS						
Residual SE	0.12	0.10	0.03	0.04	0.12	0.11
Adj. R^2	0.68	0.76	0.97	0.96	0.63	0.71
F-statistic	461.6	700.1	6789	4568	360.5	538.9
df	1, 213	1, 218	1, 216	1, 213	1, 211	1, 216
p-value	< 0.0001	< 0.0001	< 0.0001	< 0.0001	< 0.0001	< 0.0001
Slope	0.85	0.88	0.93	0.99	0.76	0.79
SE	0.04	0.03	0.01	0.01	0.04	0.03
t-value	21.5	-13.0	82.4	67.6	19.0	23.2
p-value	< 0.0001	< 0.0001	< 0.0001	< 0.0001	< 0.0001	< 0.0001
Intercept	0.47	-0.71	-0.06	1.34	0.39	-0.68
SE	0.01	0.05	0.01	0.01	0.01	0.06
t-value	32.5	26.5	-6.80	246.5	26.2	-12.1
p-value	< 0.0001	< 0.0001	< 0.0001	< 0.0001	< 0.0001	< 0.0001
SMA						
R^2	0.68	0.76	0.97	0.96	0.63	0.71
p-value	< 0.0001	< 0.0001	< 0.0001	< 0.0001	< 0.0001	< 0.0001
Slope	0.99	0.96	0.94	1.02	0.92	0.87
Limits	0.93, 1.06	0.91, 1.00	0.92, 0.96	0.98, 1.04	0.85, 0.99	0.82, 0.93
Intercept	0.44	-0.82	-0.07	1.33	0.36	-0.80
Limits	0.42, 0.47	-0.90, -0.74	-0.09, -0.05	1.32, 1.34	0.33, 0.38	-0.88, -0.71
Isometry	yes	yes		yes		
Test stat (r)	-0.02	-0.14		0.07		
df	213	218		213		
p-value	0.77	0.06		0.29		

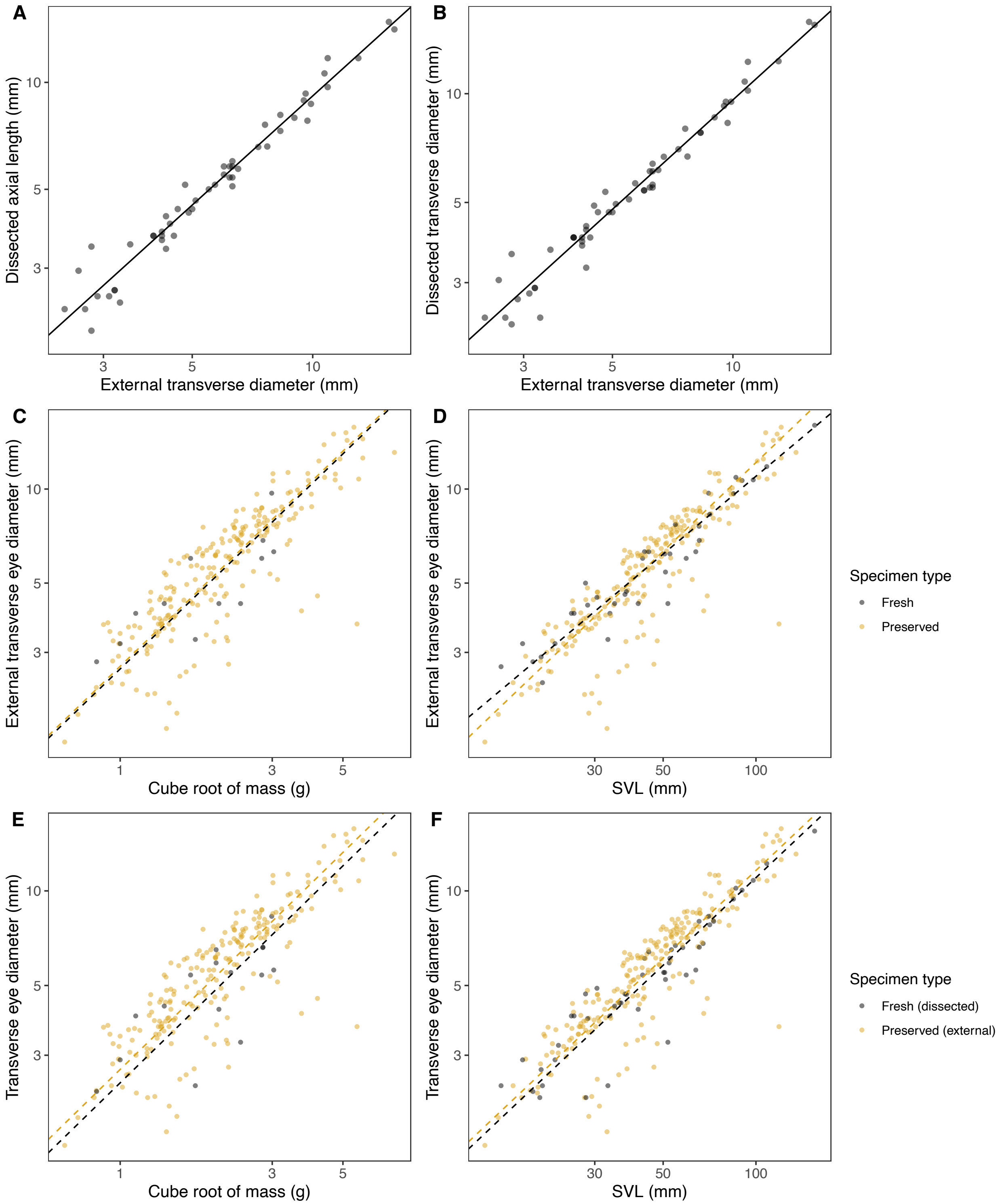

Figure S2. External measurements of anuran eye size can predict dissected measurements **(A-B)**, and eye scaling relationships derived from a large sample of preserved museum specimens yield similar fits to those derived from a smaller sample of fresh specimens **(C-F)**. **(A)** Axial eye length (AL) measured from dissected eyes is highly correlated with transverse eye diameter (ED) measured externally prior to dissection in fresh specimens (R^2 = 0.96, n = 52, $SE_{res.}$ = 0.04,

$F_{(1,50)} = 1276$, $p < 0.0001$). ED predicts AL in a log-transformed ordinary least squares regression with an allometric slope of 1.02 (SE = 0.03, t = 35.7, $p < 0.0001$) and intercept of –0.06 (SE = 0.02, t = -2.64, p = 0.01). **(B)** ED measured from dissected eyes is highly correlated with ED measured externally prior to dissection in fresh specimens ($R^2 = 0.96$, n = 55, $SE_{res.} = 0.04$, $F_{(1,53)} = 1387$, $p < 0.0001$). External ED predicts dissected ED in a log-transformed ordinary least squares regression with an allometric slope of 1.01 (SE = 0.03, t = 37.2, $p < 0.0001$) and intercept of -0.02 (SE = 0.14, t = -1.11, p = 0.27). **(C)** Externally measured ED scales with the cube root of mass (RM) allometrically via equal slopes (SMA test: Likelihood ratio stat. = 2.08, df = 1, p = 0.15) and equal intercepts (Wald stat. = 0.33, df = 1, p = 0.56) in fresh and preserved specimens. **(D)** Externally measured ED scales with snout-vent length (SVL) with unequal slopes in fresh and preserved specimens (SMA test: Likelihood ratio stat. = 5.31, df = 1, p = 0.02). The slope estimate was higher for preserved specimens (0.94) than for fresh specimens (0.83), which may indicate some shrinkage in SVL of preserved specimens. **(E)** Externally measured ED from preserved specimens scales with RM allometrically with and equal slope (SMA test: Likelihood ratio stat. = 0.43, df = 1, p = 0.51) and intercept (Wald stat. = 2.16, df = 1, p = 0.14) as ED measured from dissected eyes of fresh specimens. This indicates that using preserved specimens and external measurements provides an accurate estimate of eye scaling in anurans. **(F)** Externally measured ED from preserved specimens scales with SVL allometrically with the same slope (SMA test: Likelihood ratio stat. = 2.40, df = 1, p = 0.12) but a different intercept (Wald stat. = 7.18, df = 1, p = 0.007) than measurements of ED from dissected eyes of fresh specimens. The intercept for preserved specimens was slightly higher (-0.79) than fresh specimens (-0.81), indicating that using preserved specimens may slightly overestimate relative eye sizes but is a close approximation.

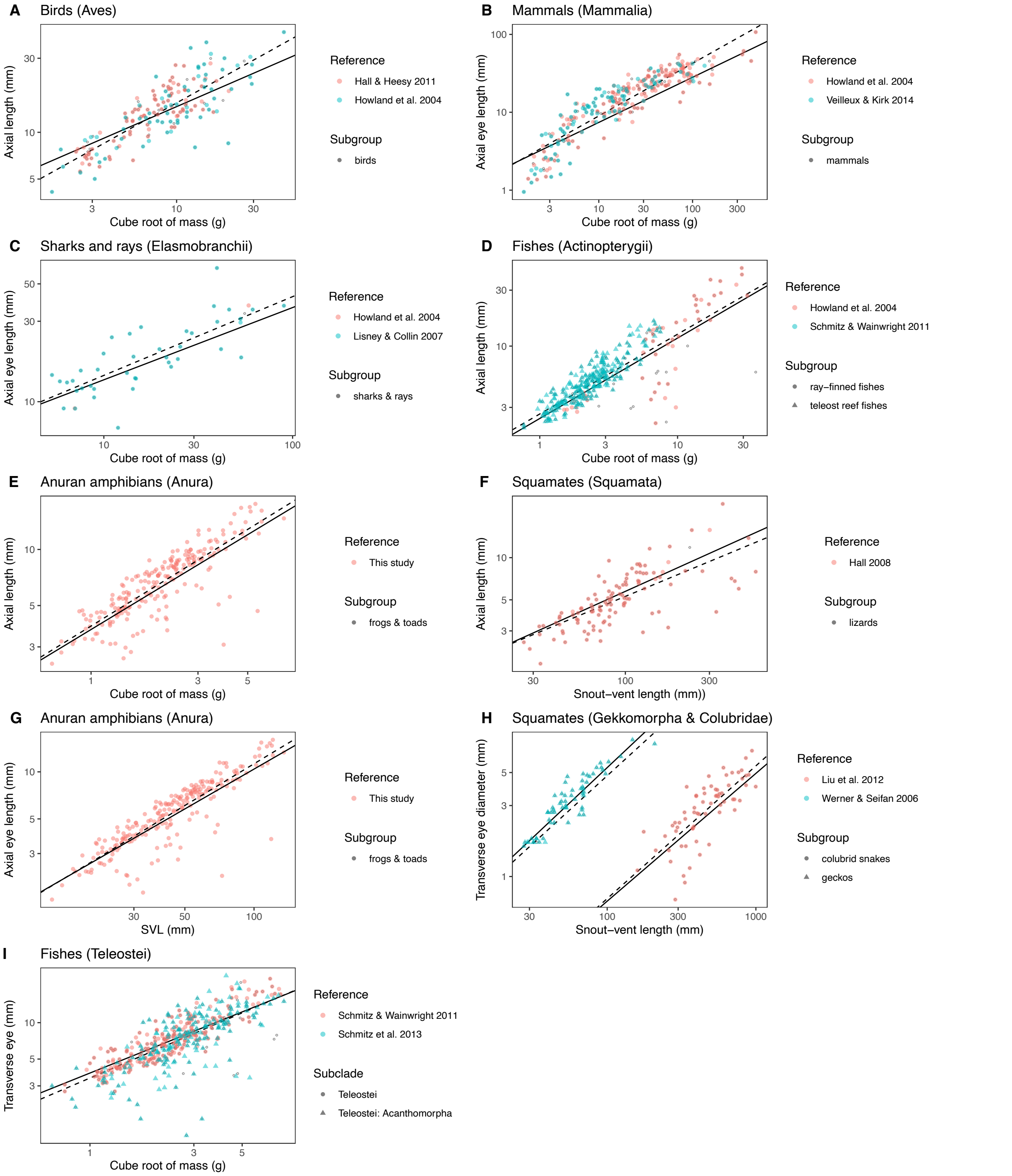

Figure S3. Compiled data for vertebrate eye and body size separated by eye measurement (axial length or transverse diameter), body size measurement (mass or snout-vent length), and vertebrate clade. Allometric scaling relationships were similar for phylogenetic generalised least squares (solid) and ordinary least squares (dashed) models.

Table S5. Phylogenetic generalised least squares (PGLS) regression fits for vertebrate eye scaling. AL = axial eye length, RM = cube root of mass, ED = transverse eye diameter, SVL = snout-vent length.

PGLS model	n	λ	95% conf. (λ)	Res. SE	Adj. R^2	F-stat.	df	p-value	Slope (a)	SE_a	t-$value_a$	p-$value_a$	Intercept (b)	SE_b	t-$value_b$	p-$value_b$
AL vs. RM																
Frogs & toads	215	0.95	0.88, 0.99	0.09	0.80	877.5	1, 213	<0.0001	0.73	0.02	29.6	<0.0001	0.57	0.05	10.8	<0.0001
Birds	139	0.88	0.67, 0.96	0.01	0.37	82.63	1, 137	<0.0001	0.45	0.05	9.09	<0.0001	0.71	0.08	9.28	<0.0001
Mammals	181	0.94	0.89, 0.97	0.02	0.58	252.4	1, 179	<0.0001	0.58	0.04	15.89	<0.0001	0.29	0.16	1.88	0.06
Ray-finned fishes	205	0.94	0.87, 0.97	0.01	0.76	631.8	1, 203	<0.0001	0.69	0.03	25.1	<0.0001	0.38	0.08	4.54	<0.0001
Sharks & rays	41	1.00	0.37, NA	0.01	0.44	32.1	1, 39	<0.0001	0.43	0.08	5.67	<0.0001	0.70	0.11	6.66	<0.0001
AL vs. SVL																
Frogs & toads	220	0.96	0.90, 1.00	0.10	0.84	1174	1, 218	<0.0001	0.85	0.02	34.3	<0.0001	-0.68	0.07	-9.75	<0.0001
Squamate lizards	106	0.63	0.35, 0.83	0.01	0.51	109.7	1, 104	<0.0001	0.57	0.05	10.5	<0.0001	-0.38	0.13	-2.91	0.004
ED vs. SVL																
Frogs & toads	220	0.96	0.90, 1.00	0.10	0.84	1177	1, 218	<0.0001	0.84	0.02	34.3	<0.0001	-0.66	0.07	-9.72	<0.0001
Squamates	127	1.00	0.79, NA	0.02	0.67	86.2	3, 123	<0.0001								
Colubrid snakes	66								0.86	0.06	13.5	<0.0001	-1.88	0.24	-7.69	<0.0001
Geckos	61								0.94	0.37	14.1	0.52	-1.15	0.60	-7.04	0.04
ED vs. RM																
Frogs & toads	215	0.96	0.90, 0.99	0.11	0.80	861.9	1, 213	<0.0001	0.82	0.03	29.4	<0.0001	0.46	0.06	7.29	<0.0001
Teleost fishes	299	0.99	0.96, 1.00	0.01	0.63	510.2	1, 297	<0.0001	0.72	0.03	22.6	<0.0001	0.58	0.06	10.2	<0.0001

Table S6. Ordinary least squares (OLS) regression fits for vertebrates. AL = axial eye length, RM = cube root of mass, ED = transverse eye diameter, SVL = snout-vent length.

OLS model	n	Res. SE	Adj. R^2	F-stat.	df	p-value	Slope (a)	SE$_a$	t-value$_a$	p-value$_a$	Intercept (b)	SE$_b$	t-value$_b$	p-value$_b$
AL vs. RM														
Frogs & toads	215	0.10	0.69	485.5	1, 213	<0.0001	0.74	0.03	22.0	<0.0001	0.59	0.01	47.3	<0.0001
Birds	139	0.12	0.65	260.9	1, 137	<0.0001	0.58	0.04	16.2	<0.0001	0.61	0.03	18.3	<0.0001
Mammals	181	0.18	0.79	689.8	1, 179	<0.0001	0.68	0.03	26.3	<0.0001	0.28	0.03	8.08	<0.0001
Ray-finned fishes	215	0.15	0.70	502	1, 213	<0.0001	0.68	0.03	22.4	<0.0001	0.42	0.02	23.9	<0.0001
Sharks & rays	41	0.13	0.60	60.5	1, 39	<0.0001	0.47	0.06	7.78	<0.0001	0.69	0.08	9.90	<0.0001
AL vs. SVL														
Frogs & toads	220	0.10	0.76	696.7	1, 218	<0.0001	0.89	0.03	26.4	<0.0001	-0.72	0.06	-13.2	<0.0001
Squamate lizards	107	0.14	0.51	111	1, 105	<0.0001	0.52	0.05	10.5	<0.0001	-0.32	0.10	-3.28	0.001
ED vs. SVL														
Frogs & toads	220	0.10	0.76	700.1	1, 218	<0.0001	0.88	0.03	26.5	<0.0001	-0.71	0.05	-13.0	<0.0001
Squamates	127	0.11	0.68	91.1	3, 123	<0.0001								
Colubrid snakes	66						0.89	0.08	11.6	<0.0001	-1.93	0.20	-9.47	<0.0001
Geckos	61						0.91	0.19	11.8	0.85	-1.09	0.45	-6.08	<0.001
ED vs. RM														
Frogs & toads	215	0.12	0.68	461.6	1, 213	<0.0001	0.85	0.04	21.5	<0.0001	0.47	0.01	32.5	<0.0001
Teleost fishes	306	0.14	0.57	406.4	1, 304	<0.0001	0.77	0.04	20.2	<0.0001	0.54	0.02	31.7	<0.0001

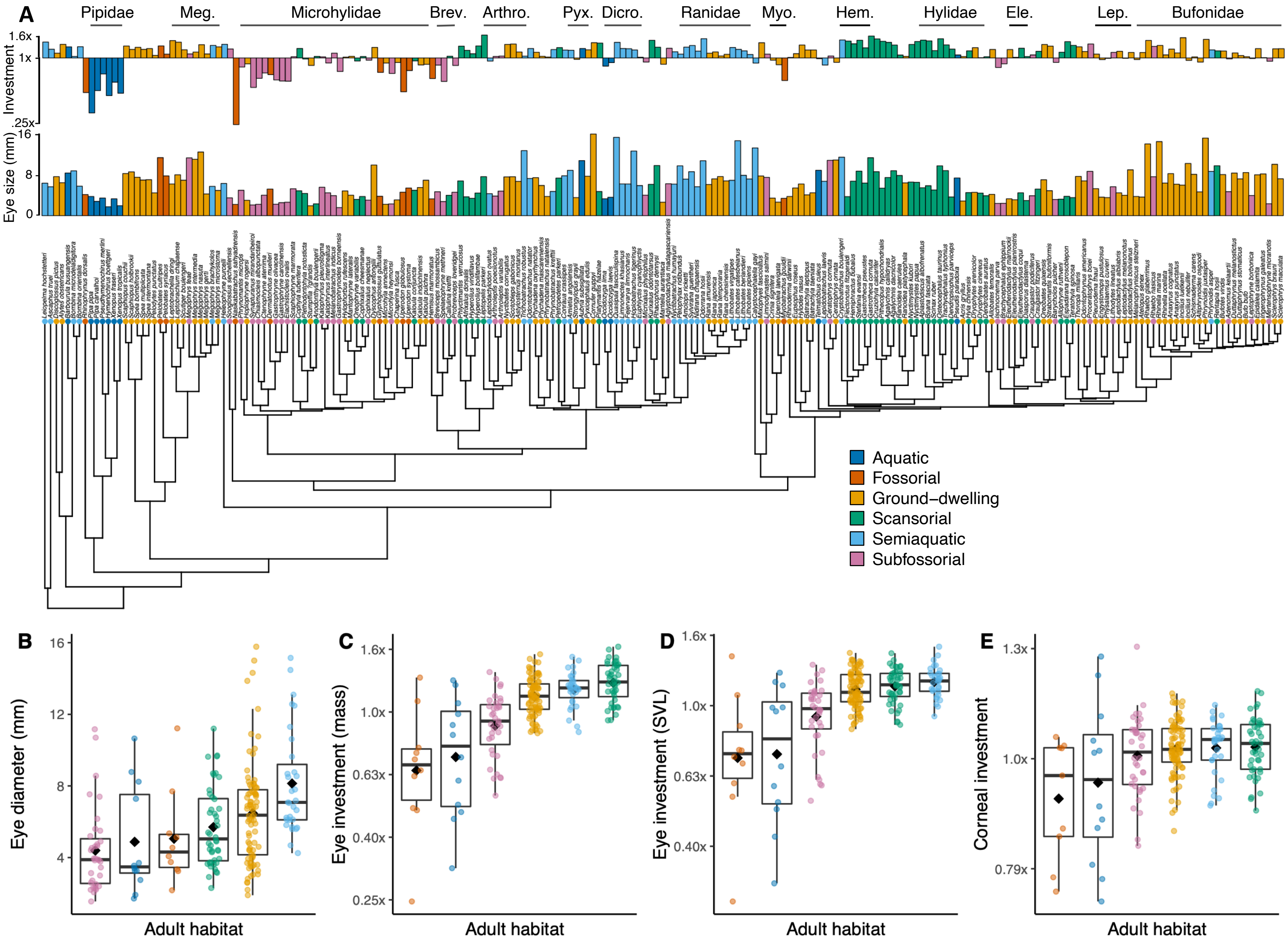
A
Pipidae
Meg.
Microhylidae
Brev.
Arthro.
Pyx.
Dicro.
Ranidae
Myo.
Hem.
Hylidae
Ele.
Lep.
Bufonidae
Investment
1.6x
1x
.25x
Eye size (mm)
16
8
0
Aquatic
Fossorial
Ground−dwelling
Scansorial
Semiaquatic
Subfossorial
B
Eye diameter (mm)
16
12
8
4
Adult habitat
C
Eye investment (mass)
1.6x
1.0x
0.63x
0.40x
0.25x
Adult habitat
D
Eye investment (SVL)
1.6x
1.0x
0.63x
0.40x
Adult habitat
E
Corneal investment
1.3x
1.0x
0.79x
Adult habitat

Figure S4. Anuran eye size, eye investment relative to mass, eye investment relative to snout-vent length (SVL), and corneal investment relative to eye size across adult habitats. **(A)** Phylogeny adapted from [36] showing species means for both absolute eye diameter and eye investment relative to body mass across the phylogeny. Bars and phylogeny tips are colored by mating habitat. Meg. = Megophryidae, Brev. = Brevicepitidae, Arthro. = Arthroleptidae, Pyx. = Pyxicephalidae, Dicro. = Dicroglossidae, Myo. = Myobatrachidae, Hem. = Hemiphractidae, Ele. = Eleutherodactylidae, Lep. = Leptodactylidae. **(B)** Absolute eye size, **(C)** eye investment relative to mass, **(D)** eye investment relative to SVL and **(E)** cornea investment relative to eye size. Black diamonds indicate the means and black bars the medians of each adult habitat state. Kruskal-Wallis tests treating species as independent showed significant differences in absolute eye size, eye investment relative to mass, and eye investment relative to SVL, but not cornea investment relative to eye size among adult habitats. Phylogenetic ANCOVAs indicated that habitat has significant effects on eye size relative to mass, eye size relative to SVL, and cornea size relative to eye size.

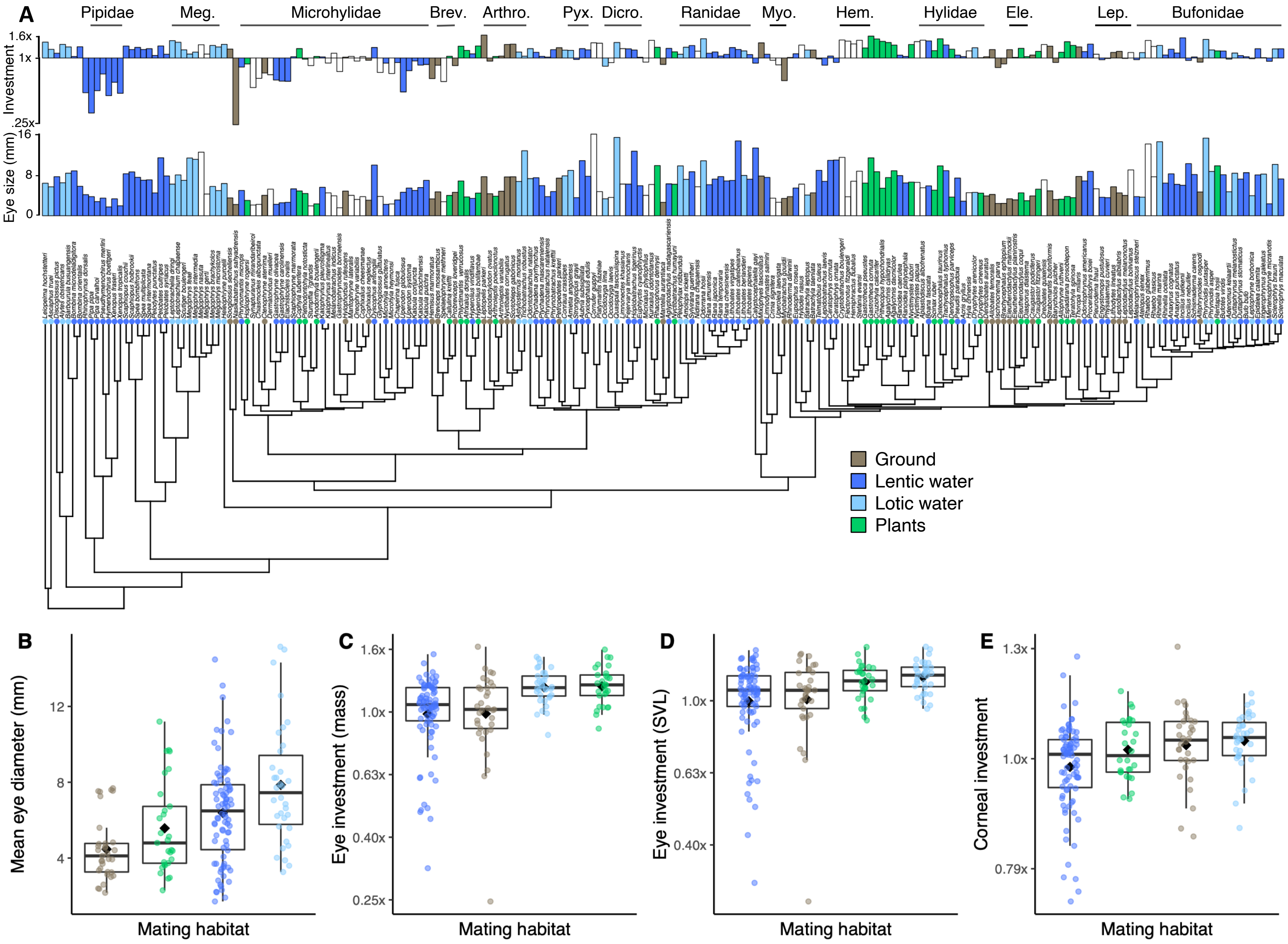
A
Pipidae
Meg.
Microhylidae
Brev.
Arthro.
Pyx.
Dicro.
Ranidae
Myo.
Hem.
Hylidae
Ele.
Lep.
Bufonidae
Investment
1.6x
1x
.25x
Eye size (mm)
16
8
0
Ground
Lentic water
Lotic water
Plants
B
Mean eye diameter (mm)
12
8
4
Mating habitat
C
Eye investment (mass)
1.6x
1.0x
0.63x
0.40x
0.25x
Mating habitat
D
Eye investment (SVL)
1.0x
0.63x
0.40x
Mating habitat
E
Corneal investment
1.3x
1.0x
0.79x
Mating habitat

Figure S5. Anuran eye size, eye investment relative to mass, eye investment relative to snout-vent length (SVL), and corneal investment relative to eye size across mating habitats. **(A)** Phylogeny adapted from [36] showing species means for both absolute eye diameter and eye investment relative to body mass across the phylogeny. Bars and phylogeny tips are colored by mating habitat. Meg. = Megophryidae, Brev. = Brevicepitidae, Arthro. = Arthroleptidae, Pyx. = Pyxicephalidae, Dicro. = Dicroglossidae, Myo. = Myobatrachidae, Hem. = Hemiphractidae, Ele. = Eleutherodactylidae, Lep. = Leptodactylidae. **(B)** Absolute eye size, **(C)** eye investment relative to mass, **(D)** eye investment relative to SVL and **(E)** cornea investment relative to eye size. Black diamonds indicate the means and black bars the medians of each mating habitat state. Kruskal-Wallis tests treating species as independent showed significant differences in absolute eye size, eye investment relative to mass, eye investment relative to SVL, and cornea investment relative to eye size among mating habitats. Phylogenetic ANCOVAs indicated that mating habitat has significant effects on eye size relative to mass, eye size relative to SVL, and cornea size relative to eye size.

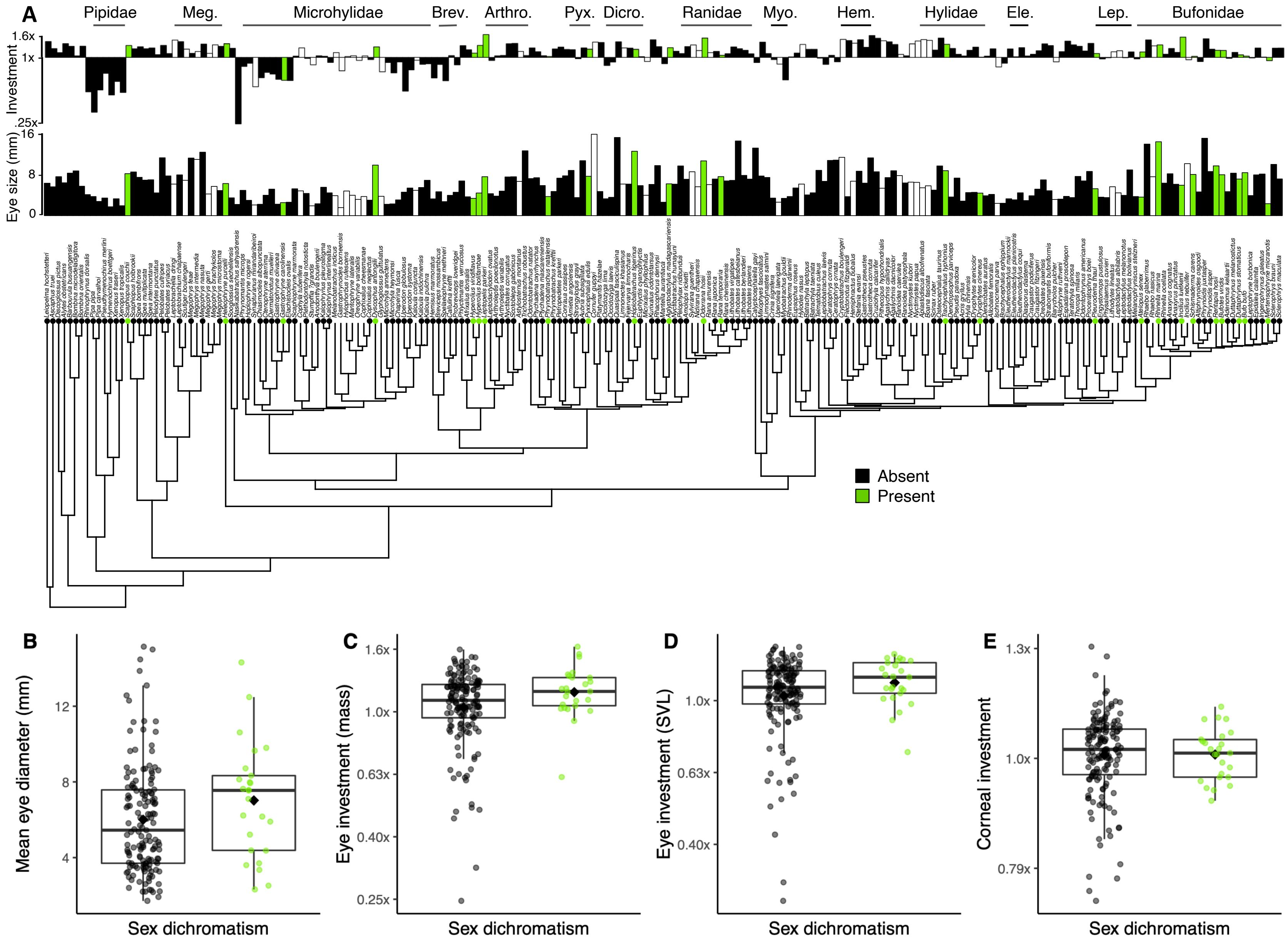
A
Pipidae
Meg.
Microhylidae
Brev.
Arthro.
Pyx.
Dicro.
Ranidae
Myo.
Hem.
Hylidae
Ele.
Lep.
Bufonidae
Investment
1.6x
1x
.25x
Eye size (mm)
16
8
0
Absent
Present
B
Mean eye diameter (mm)
12
8
4
Sex dichromatism
C
Eye investment (mass)
1.6x
1.0x
0.63x
0.40x
0.25x
Sex dichromatism
D
Eye investment (SVL)
1.0x
0.63x
0.40x
Sex dichromatism
E
Corneal investment
1.3x
1.0x
0.79x
Sex dichromatism

Figure S6. Anuran eye size, eye investment relative to mass, eye investment relative to snout-vent length (SVL), and corneal investment relative to eye size across sexual dichromatism states. **(A)** Phylogeny adapted from [36] showing species means for both absolute eye diameter and phylogenetically corrected eye investment relative to body mass across the phylogeny. Bars and phylogeny tips are colored by the presence or absence of sexual dichromatism. Meg. = Megophryidae, Brev. = Brevicepitidae, Arthro. = Arthroleptidae, Pyx. = Pyxicephalidae, Dicro. = Dicroglossidae, Myo. = Myobatrachidae, Hem. = Hemiphractidae, Ele. = Eleutherodactylidae, Lep. = Leptodactylidae. **(B)** Absolute eye size, **(C)** eye investment relative to mass, **(D)** eye investment relative to SVL and **(E)** cornea investment relative to eye size. Black diamonds indicate the means and black bars the medians of each sexual dichromatism state. Kruskal-Wallis tests treating species as independent showed no significant differences in absolute eye size, eye investment relative to mass, eye investment relative to SVL, or cornea investment relative to eye size among sexual dichromatism states. Phylogenetic ANCOVAs indicated that sexual dichromatism has no significant effects on eye size relative to mass, eye size relative to SVL, or cornea size relative to eye size.

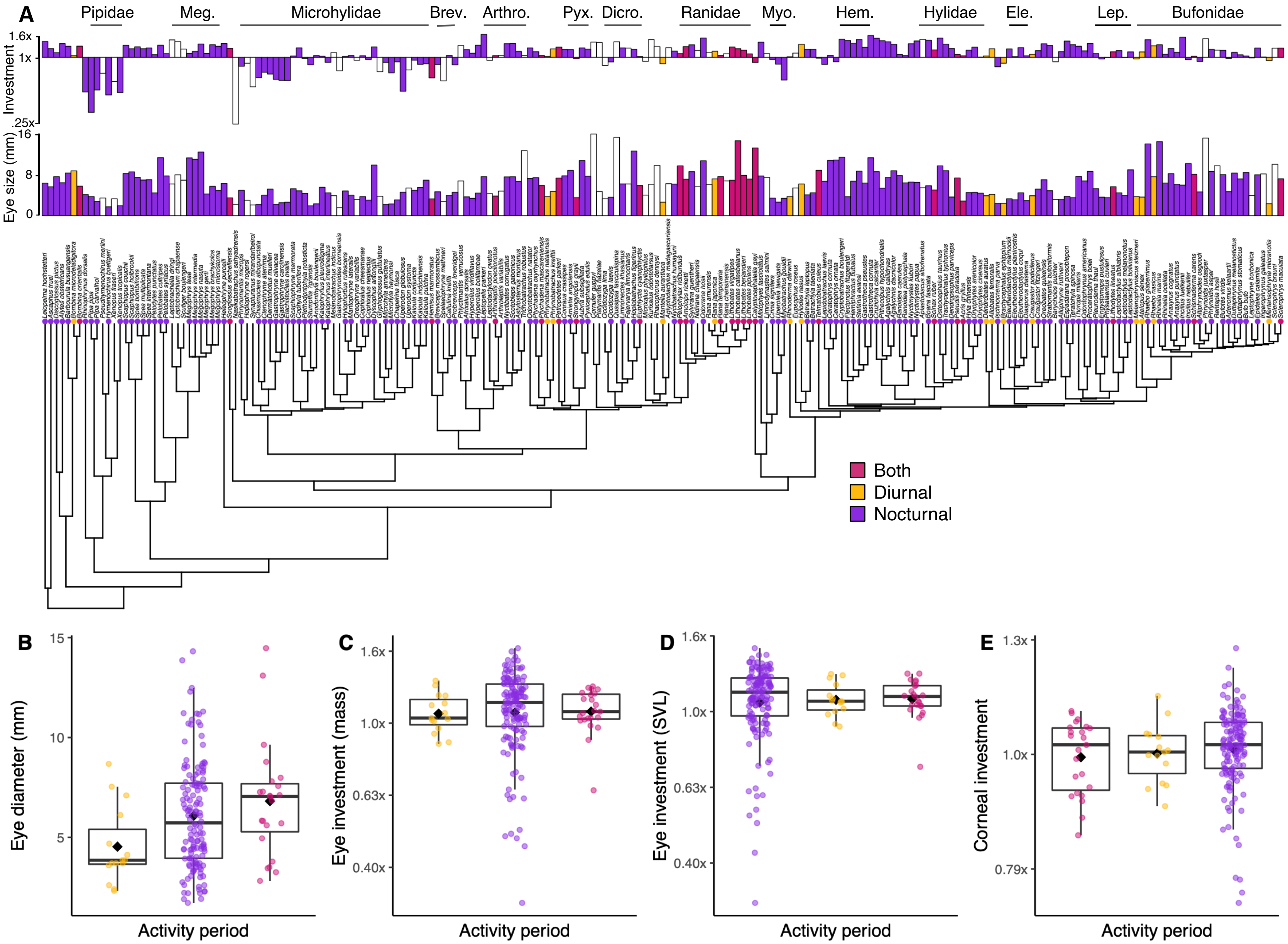
A
Pipidae
Meg.
Microhylidae
Brev.
Arthro.
Pyx.
Dicro.
Ranidae
Myo.
Hem.
Hylidae
Ele.
Lep.
Bufonidae
Investment
1.6x
1x
.25x
Eye size (mm)
16
8
0
Both
Diurnal
Nocturnal
B
Eye diameter (mm)
15
10
5
Activity period
C
Eye investment (mass)
1.6x
1.0x
0.63x
0.40x
Activity period
D
Eye investment (SVL)
1.6x
1.0x
0.63x
0.40x
Activity period
E
Corneal investment
1.3x
1.0x
0.79x
Activity period

Figure S7. Anuran eye size, eye investment relative to mass, eye investment relative to snout-vent length (SVL), and corneal investment relative to eye size across adult activity patterns. **(A)** Phylogeny adapted from [36] showing species means for both absolute eye diameter and eye investment relative to body mass across the phylogeny. Bars and phylogeny tips are colored by activity pattern. Meg. = Megophryidae, Brev. = Brevicepitidae, Arthro. = Arthroleptidae, Pyx. = Pyxicephalidae, Dicro. = Dicroglossidae, Myo. = Myobatrachidae, Hem. = Hemiphractidae, Ele. = Eleutherodactylidae, Lep. = Leptodactylidae. **(B)** Absolute eye size, **(C)** eye investment relative to mass, **(D)** eye investment relative to SVL and **(E)** cornea investment relative to eye size. Black diamonds indicate the means and black bars the medians of each activity pattern state. Kruskal-Wallis tests treating species as independent showed significant differences in absolute eye size, but no significant differences in eye investment relative to mass, eye investment relative to SVL, or cornea investment relative to eye size among activity patterns. Phylogenetic ANCOVAs indicated that activity pattern has significant effects on eye size relative to mass, eye size relative to SVL, and cornea size relative to eye size.

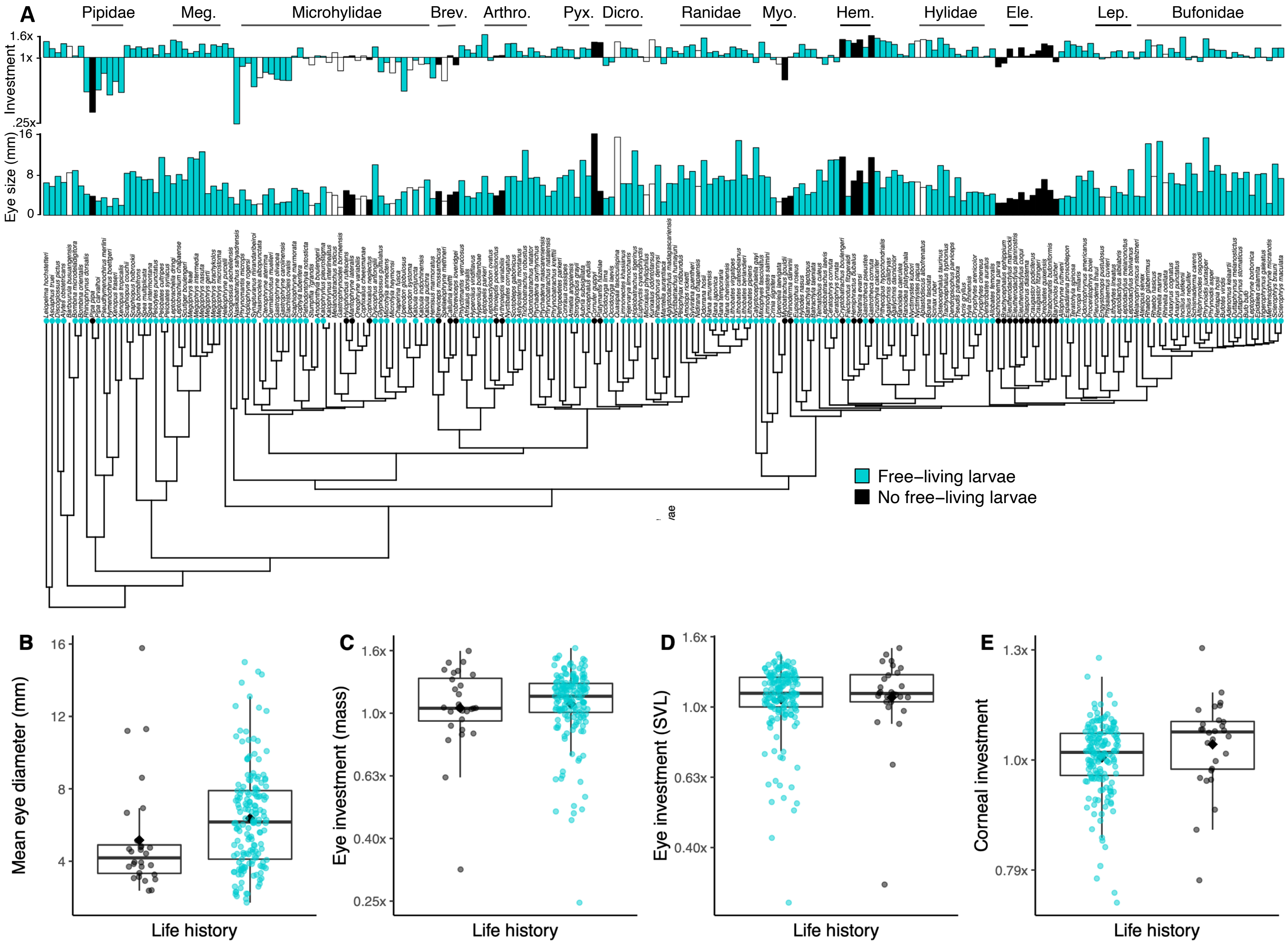
A
Pipidae
Meg.
Microhylidae
Brev.
Arthro.
Pyx.
Dicro.
Ranidae
Myo.
Hem.
Hylidae
Ele.
Lep.
Bufonidae
Investment
1.6x
1x
.25x
Eye size (mm)
16
8
0
Free–living larvae
No free–living larvae
B
Mean eye diameter (mm)
Life history
C
Eye investment (mass)
Life history
D
Eye investment (SVL)
Life history
E
Corneal investment
Life history

Figure S8. Anuran eye size, eye investment relative to mass, eye investment relative to snout-vent length (SVL), and corneal investment relative to eye size in anurans with and without free-living larvae. **(A)** Phylogeny adapted from [36] showing species means for both absolute eye diameter and eye investment relative to body mass across the phylogeny. Bars and phylogeny tips are colored by life history strategy. Meg. = Megophryidae, Brev. = Brevicepitidae, Arthro. = Arthroleptidae, Pyx. = Pyxicephalidae, Dicro. = Dicroglossidae, Myo. = Myobatrachidae, Hem. = Hemiphractidae, Ele. = Eleutherodactylidae, Lep. = Leptodactylidae. **(B)** Absolute eye size, **(C)** eye investment relative to mass, **(D)** eye investment relative to SVL and **(E)** cornea investment relative to eye size. Black diamonds indicate the means and black bars the medians of each life history state. Kruskal-Wallis tests treating species as independent showed significant differences in absolute eye size and cornea investment relative to eye size, but no significant difference in eye investment relative to mass or eye investment relative to SVL among life history strategies. Phylogenetic ANCOVAs indicated that life history has no significant effects on eye size relative to mass, eye size relative to SVL, or cornea size relative to eye size.

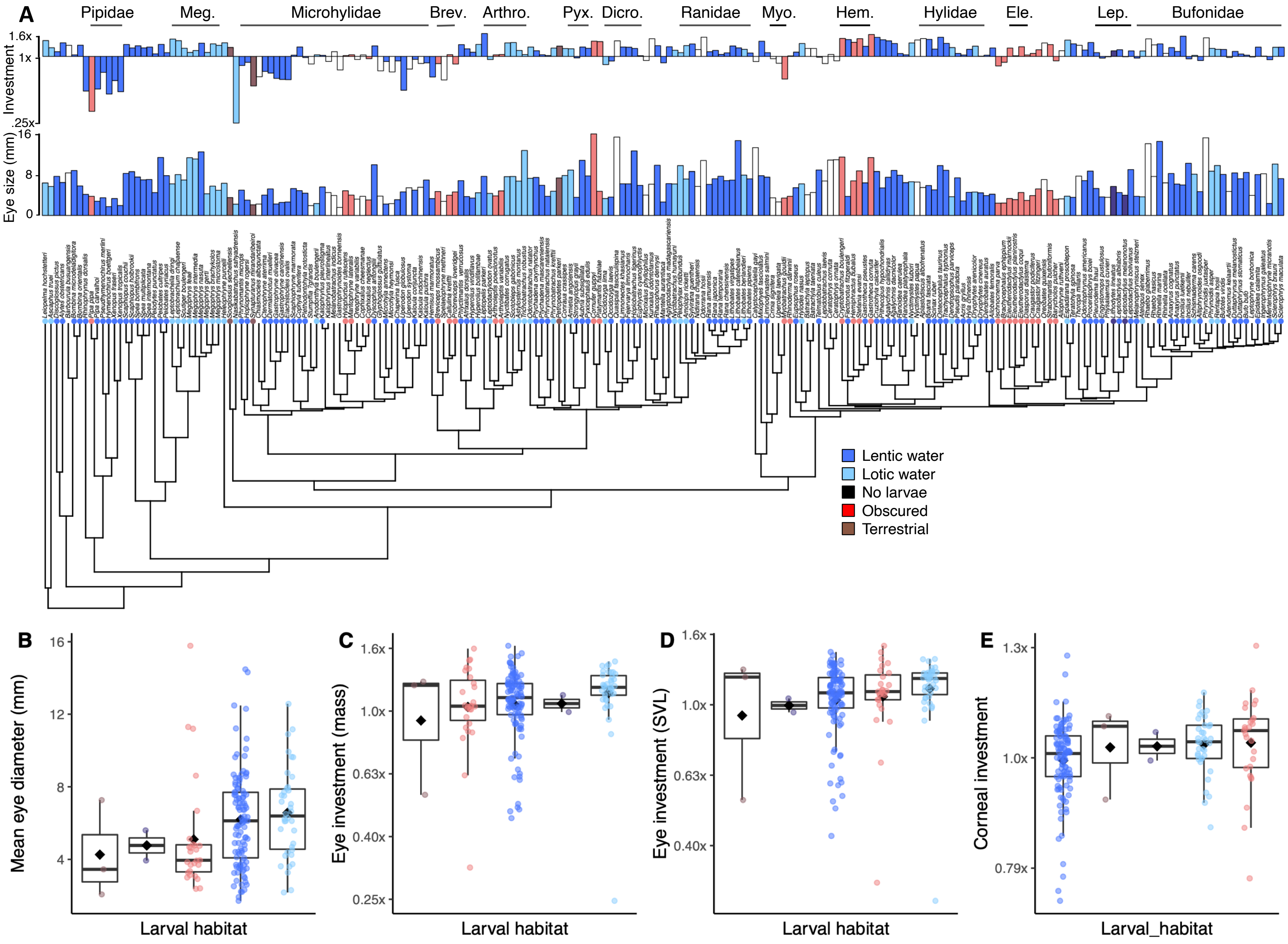
A
Pipidae
Meg.
Microhylidae
Brev.
Arthro.
Pyx.
Dicro.
Ranidae
Myo.
Hem.
Hylidae
Ele.
Lep.
Bufonidae
Investment
1.6x
1x
.25x
Eye size (mm)
16
8
0
Lentic water
Lotic water
No larvae
Obscured
Terrestrial
B
Mean eye diameter (mm)
Larval habitat
C
Eye investment (mass)
Larval habitat
D
Eye investment (SVL)
Larval habitat
E
Corneal investment
Larval_habitat

Figure S9. Anuran eye size, eye investment relative to mass, eye investment relative to snout-vent length (SVL), and corneal investment relative to eye size across larval habitats. **(A)** Phylogeny adapted from [36] showing species means for both absolute eye diameter and eye investment relative to body mass across the phylogeny. Bars and phylogeny tips are colored by mating habitat. Meg. = Megophryidae, Brev. = Brevicepitidae, Arthro. = Arthroleptidae, Pyx. = Pyxicephalidae, Dicro. = Dicroglossidae, Myo. = Myobatrachidae, Hem. = Hemiphractidae, Ele. = Eleutherodactylidae, Lep. = Leptodactylidae. **(B)** Absolute eye size, **(C)** eye investment relative to mass, **(D)** eye investment relative to SVL and **(E)** cornea investment relative to eye size. Black diamonds indicate the means and black bars the medians of each larval habitat state. Kruskal-Wallis tests treating species as independent showed significant differences in absolute eye size and cornea investment relative to eye size, but no significant difference in eye investment relative to mass or eye investment relative to SVL among larval habitats. Phylogenetic ANCOVAs indicated that larval habitat has no significant effects on eye size relative to mass, eye size relative to SVL, or cornea size relative to eye size.

Table S7. Results of non-parametric Kruskal-Wallis tests for differences in absolute eye diameters, eye investments relative to body mass, eye investments relative to snout-vent length, and corneal investments relative to eye diameter across states for six ecological traits. Eye investments are corrected for phylogeny and allometry with body size; cornea investments are corrected for phylogeny and allometry with eye size; species are treated as independent. Significant results are bolded.

	Ecological factor					
**Metric**	**Adult habitat**	**Activity period**	**Mating habitat**	**Sexual dichromatism**	**Life history**	**Larval habitat**
Eye diameter						
χ^2	36.3	6.37	24.6	3.20	6.94	10.5
df	5	2	3	1	1	4
p-value	**<0.0001**	**0.04**	**<0.0001**	0.07	**0.008**	**0.03**
Eye investment (mass)						
χ^2	68.3	2.07	28.6	3.19	0.50	7.89
df	5	2	3	1	1	4
p-value	**<0.0001**	0.36	**<0.0001**	0.07	0.48	0.10
Eye investment (SVL)						
χ^2	49.9	0.95	14.6	2.17	0.28	7.5
df	5	2	3	1	1	4
p-value	**<0.0001**	0.62	**0.002**	0.14	0.60	0.11
Corneal investment						
χ^2	8.95	1.78	15.1	0.33	4.41	10.4
df	5	2	3	1	1	4
p-value	0.11	0.41	**0.002**	0.57	**0.04**	**0.03**

Table S8. Results of phylogenetic generalised least squares (PGLS) models of transverse eye diameter (ED) vs. each ecological trait with the cube root of mass (RM) as a covariate. Significant effects are bolded. No individual trait states or interactions in coefficients were significant except in the model including mating habitat, which showed a positive interaction between RM and the lotic water mating habitat (p = 0.04) and a positive interaction between RM and the plants mating habitat (0.03).

**Model/factors**	**DF**	**Sum Sq.**	**Mean Sq.**	**F-value**	**p-value**
ED ~ RM * Adult habitat					
RM	1, 203	7.70	7.70	1037.6	**<0.0001**
Adult habitat	5, 203	0.46	0.09	12.38	**<0.0001**
RM x Adult habitat	5, 203	0.09	0.02	2.30	**0.046**
ED ~ RM * Activity period					
RM	1, 170	6.21	6.21	754.7	**<0.0001**
Activity period	2, 170	0.82	0.41	49.6	**<0.0001**
RM x Activity period	2, 170	0.005	0.002	0.29	0.75
ED ~ RM * Mating habitat					
RM	1, 170	7.33	7.33	665.1	**<0.0001**
Mating habitat	3, 170	0.12	0.04	3.71	**0.01**
RM x Mating habitat	3, 170	0.07	0.02	2.08	0.10
ED ~ RM * Sexual dichromatism					
RM	1, 172	8.37	8.37	679.4	**<0.0001**
Sexual dichromatism	1, 172	0.007	0.007	0.55	0.46
RM x Sexual dichromatism	1, 172	0.01	0.01	1.12	0.29
ED ~ RM * Life history					
RM	1, 189	9.27	9.27	761.1	**<0.0001**
Life history	1, 189	0.01	0.01	1.00	0.32
RM x Life history	1, 189	0.001	0.001	0.11	0.74
ED ~ RM * Larval habitat					
RM	1, 166	8.99	8.99	692.3	**<0.0001**
Larval habitat	4, 166	0.05	0.01	1.05	0.38
RM x Larval habitat	4, 166	0.06	0.01	1.13	0.34

Table S9. Results of phylogenetic generalised least squares (PGLS) models of transverse eye diameter (ED) vs. each ecological trait with the cube root of mass (SVL) as a covariate. Significant effects are bolded.

Model/factors	DF	Sum Sq.	Mean Sq.	F-value	p-value
ED ~ SVL * Adult habitat					
SVL	1, 208	9.93	9.93	1339.5	**<0.0001**
Adult habitat	5, 208	0.28	0.06	7.58	**<0.0001**
SVL x Adult habitat	5, 208	0.06	0.01	1.63	0.15
ED ~ SVL * Activity period					
SVL	2, 173	0.77	0.38	61.8	**<0.0001**
Activity period	1, 173	6.29	6.29	1013.9	**<0.0001**
SVL x Activity period	2, 173	0.0022	0.0011	0.18	0.84
ED ~ SVL * Mating habitat					
SVL	1, 172	7.82	7.82	891.34	**<0.0001**
Mating habitat	3, 172	0.06	0.02	2.24	0.09
SVL x Mating habitat	3, 172	0.03	0.01	1.27	0.28
ED ~ SVL * Sexual dichromatism					
SVL	1, 173	8.18	8.18	865.15	**<0.0001**
Sexual dichromatism	1, 173	0.001	0.001	0.15	0.70
SVL x Sexual dichromatism	1, 173	0.01	0.01	1.51	0.22
ED ~ SVL * Life history					
SVL	1, 191	10.4	10.4	1065.0	**<0.0001**
Life history	1, 191	0.01	0.01	1.00	0.32
SVL x Life history	1, 191	0.01	0.01	1.34	0.25
ED ~ SVL * Larval habitat					
SVL	1, 168	9.37	9.37	922.2	**<0.0001**
Larval habitat	4, 168	0.03	0.008	0.75	0.56
SVL x Larval habitat	4, 168	0.10	0.02	2.37	0.05

Table S10. Results of phylogenetic generalised least squares (PGLS) models of transverse cornea diameter (CD) vs. each ecological trait, with transverse eye diameter (ED) as a covariate. Significant effects are bolded.

Model/factors	DF	Sum Sq.	Mean Sq.	F-value	p-value
CD ~ ED * Adult habitat					
ED	1, 206	3.7	3.7	7694.9	**<0.0001**
Adult habitat	5, 206	0.01	0.003	5.14	**0.0002**
ED x Adult habitat	5, 206	0.006	0.001	2.63	**0.03**
CD ~ ED * Activity period					
ED	1, 172	2.67	2.67	4921.2	**<0.0001**
Activity period	2, 172	0.10	0.05	96.67	**<0.0001**
ED x Activity period	2, 172	<0.001	0.0001	0.34	0.71
CD ~ ED * Mating habitat					
ED	1, 170	2.75	2.75	4893.6	**<0.0001**
Mating habitat	3, 170	0.009	0.003	5.15	**0.002**
ED x Mating habitat	3, 170	0.0007	0.0002	0.42	0.74
CD ~ ED * Sexual dichromatism					
ED	1, 171	2.93	2.93	4880.9	**<0.0001**
Sexual dichromatism	1, 171	0.00001	0.00001	0.01	0.91
ED x Sexual dichromatism	1, 171	0.00005	0.00005	0.08	0.78
CD ~ ED * Life history					
ED	1, 189	3.29	3.29	4653.1	**<0.0001**
Life history	1, 189	0.001	0.001	1.65	0.20
ED x Life history	1, 189	0.0006	0.0006	0.80	0.37
CD ~ ED * Larval habitat					
ED	1, 166	3.01	3.01	3985.8	**<0.0001**
Larval habitat	4, 166	0.002	0.0005	0.60	0.66
ED x Larval habitat	4, 166	0.005	0.001	1.65	0.16

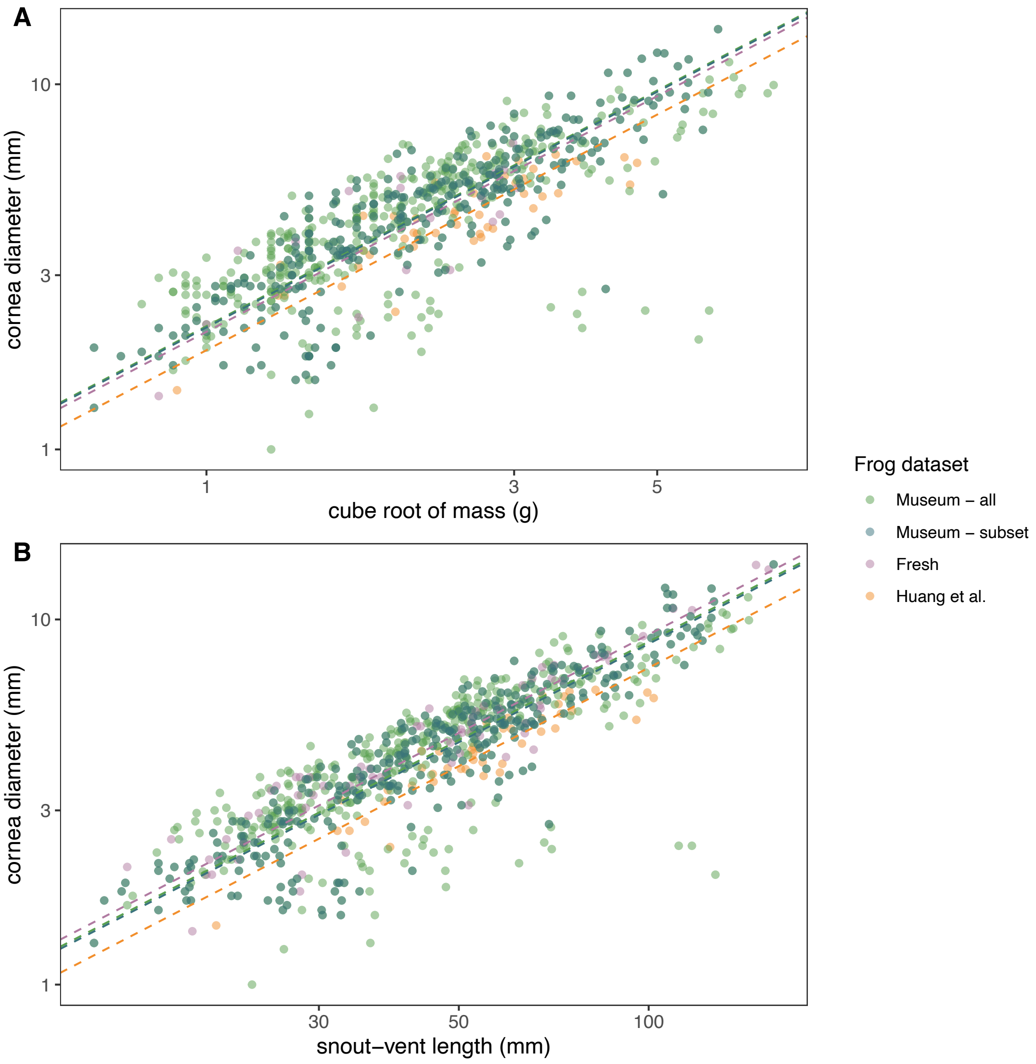

Figure S10. Comparison of our data with those from a paper measuring cornea-body size allometry in fresh frogs [28]. We compared our full museum specimen dataset, a subset of our museum data matching the families sampled in the other study, our fresh specimen dataset, and the fresh specimen dataset from the other study with standardized major axis (SMA) tests for differences in slopes and intercepts. **(A)** Corneal scaling with the cube root of mass shows no difference in slopes among datasets (Likelihood ratio stat.: 2.52, df = 3, $p = 0.47$) but does show a significant difference in intercepts (Wald stat: 46.8, df = 3, $p < 0.0001$). **(B)** Corneal scaling with snout-vent length (SVL) shows no difference in slopes among datasets (Likelihood ratio stat.: 1.90, df = 3, $p = 0.59$) but a significant difference in intercepts (Wald stat.: 102.6, df = 3, $p < 0.0001$).

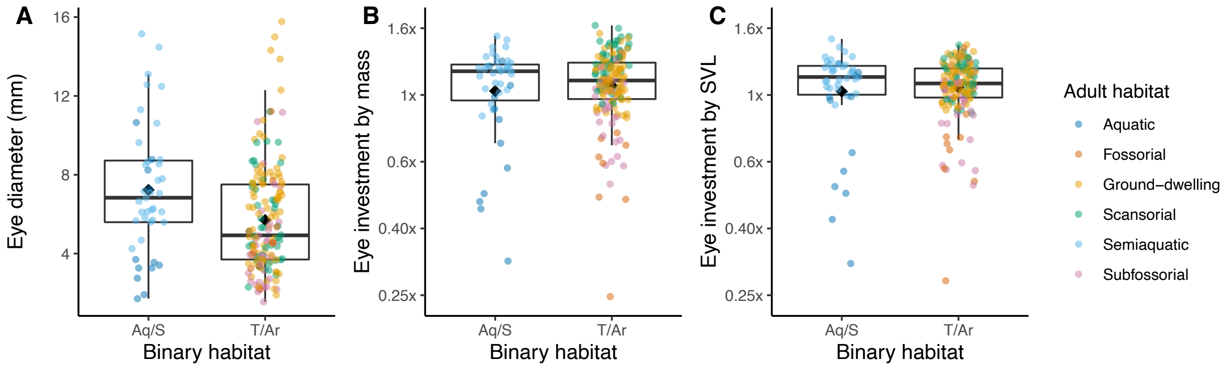

Figure S11. Recategorization of our species data into the binary habitat states used by another study of frog corneal size [28] replicates their finding that relative eye (corneal) size/investment does not differ across habitats. **(A)** Absolute eye diameter is significantly different across binary habitat states (χ^2 = 8.18, df = 1, p = 0.004). **(B)** Eye investment relative to mass (χ^2 = 0.052, df = 1, p = 0.82) and **(C)** eye investment relative to SVL (χ^2 = 8.18, df = 1, p = 0.36) do not significantly differ across binary habitat states. Aq/S = aquatic and semiaquatic species and T/Ar = terrestrial and arboreal species according to states defined by Huang et al [28]. Black bars indicate the medians and black diamonds the means for each state. Points are colored by the finer-resolution adult habitat categorizations we used in this study.

Supplemental References

Full references used in assigning each anuran species to ecological traits/states (Table S1)

Adams, S. B., and C. A. Frissell. 2001. Thermal habitat use and evidence of seasonal migration by Rocky Mountain Tailed Frogs, *Ascaphus montanus*, in Montana. Can. Field-Naturalist 115:251–256.

Alcala, A. C., and W. C. Brown. 1987. Notes on the microhabitats of the Phillipine discoglossid frog *Barbourula busuangensis*. Silliman J. 34:12–17.

Amiet, J.-L. 2012. Les Rainettes du Cameroun: (Amphibiens Anoures). Jean-Louis Amiet & la Nef des livres, Saint-Nazaire, France.

Amphibians and reptiles of peninsular Malaysia. 2009. <www.amphibia.my>

AmphibiaWeb. 2019. <www.amphibiaweb.com>

Anderson, S. R., and J. J. Wiens. 2017. Out of the dark: 350 million years of conservatism and evolution in diel activity patterns in vertebrates. Evolution 71:1944–1959.

Anfibios del Ecuador. 2019. <https://bioweb.bio/faunaweb/amphibiaweb/>

Anstis, M. 2017. Tadpoles and frogs of Australia. Second Edi. New Holland Publishers, London, Sydney, Auckland.

Anstis, M., L. C. Price, J. D. Roberts, S. R. Catalano, H. B. Hines, P. Doughty, and S. C. Donnellan. 2016. Revision of the water-holding frogs, *Cyclorana platycephala* (Anura: Hylidae), from arid Australia, including a description of a new species. Zootaxa 4126:451–479.

Arnold, E. N. 2002. A field guide to the reptiles and amphibians of Britain and Europe. Collins, London, UK.

Bain, R. H., and N. Q. Truong. 2006. Three new species of Narrow-Mouth Frogs (Genus: *Microhyla*) from Indochina, with comments on *Microhyla annamensis* and *Microhyla palmipes*. Copeia 2004:507–524.

Bain, R. H., N. Q. Truong, and D. Van Kien. 2007. New herpetofaunal records from Vietnam. Herpetol. Rev. 38:107–117.

Barbour, T. and A. Loveridge. 1928. A comparative study of the herpetological faunae of the Ulguru and Usambara Mountains, Tanganyika Territory with descriptions of new species. Mem. Museum Comp. Zool. 50:87–265.

Barej, M. F., M.-O. Rödel, L. N. Gonwouo, O. S. G. Pauwels, W. Böhme, and A. Schmitz. 2010. Review of the genus *Petropedetes* Reichenow, 1874 in Central Africa with the description of three new species (Amphibia: Anura: Petropedetidae). Zootaxa 2340:1–49.

Barej, M., A. Schmitz, J. Penner, J. Doumbia, L. Sandberger-Loua, M. Hirschfeld, C. Brede, M. Emmrich, N. G. Kouamé, A. Hillers, N. L. Gonwouo, J. Nopper, P. J. Adeba, M. A. Bangoura, C. Gage, G. Anderson, and M.-O. Rödel. 2015. Life in the spray zone – overlooked diversity in West African torrent-frogs (Anura, Odontobatrachidae, *Odontobatrachus*). Zoosystematics Evol. 91:115–149.

Behrens, K., and K. Barnes. 2016. Wildlife of Madagascar. Princeton University Press, New Jersey.

Bell, B. D. 1978. Observations on the ecology and reproduction of the New Zealand leiopelmid frogs. Herpetologica 34:340–354.

Bell, B. D., and R. J. Wassersug. 2003. Anatomical features of *Leiopelma* embryos and larvae: Implications for anuran evolution. J. Morphol. 256:160–170.

Bell, R. C., G. N. Webster, and M. J. Whiting. 2017. Breeding biology and the evolution of dynamic sexual dichromatism in frogs. J. Evol. Biol. 30:2104–2115.

Bell, R. C., and K. R. Zamudio. 2012. Sexual dichromatism in frogs: Natural selection, sexual selection and unexpected diversity. Proc. R. Soc. B Biol. Sci. 279:4687–4693.

Biju, S. D., I. Van Bocxlaer, S. Mahony, K. P. Dinesh, C. Radhakrishnan, A. Zachariah, V. Giri, and F. Bossuyt. 2011. A taxonomic review of the Night Frog genus *Nyctibatrachus* Boulenger, 1882 in the Western Ghats, India (Anura: Nyctibatrachidae) with description of twelve new species. Zootaxa 3029:1–96.

Blackburn, D. C., C. D. Siler, A. C. Diesmos, J. A. Mcguire, D. C. Cannatella, and R. M. Brown. 2013. An adaptive radiation of frogs in a Southeast Asian island archipelago. Evolution 67:2631–2646.

Brown, W. C., R. M. Brown, and A. Alcala. 1997. Species of the hazelae group of *Platymantis* (Amphibia: Ranidae) from the Philippines, with descriptions of two new species. Proc. Calif. Acad. Sci. 49:405–421.

Caldwell, J. P. and M. S. Hoogmoed. 1998. Allophrynidae, *Allophryne*, *A. ruthveni*. Cat. Am. Amphib. Reptil. 666.1-666.3.

Chan, K. O., D. C. Blackburn, R. W. Murphy, B. L. Stuart, D. A. Emmett, C. T. Ho, and R. M. Brown. 2013. A new species of narrow-mouthed frog of the genus *Kaloula* from eastern

Indochina. Herpetologica 69:329–341.

Chanda, S. K. 2002. Handbook of Indian amphibians. Director, Zoological Survey of India, Calcutta.

Channing, A. 2001. Amphibians of central and southern Africa. Cornell University Press, Ithaca, NY, USA.

Channing, A. and K. M. Howell. 2006. Amphibians of East Africa. Cornell University Press, Ithaca, NY, USA.

Channing, A., M.-O. Rödel, and J. Channing. 2012. Tadpoles of Africa. The biology and identification of all known tadpoles of Sub-Saharan Africa. Edition Chimaira, Frankfurt-am-Main.

Church, G. 1960. The Invasion of Bali by *Bufo melanostictus*. Herpetologica 16:15–21.

Cogălniceanu, D., D. Roşioru, P. Székely, D. Székely, E. Buhaciuc, F. Stănescu, and C. Miaud. 2015. Age and body size in populations of two syntopic spadefoot Toads (Genus *Pelobates*) at the limit of their ranges. J. Herpetol. 48:537–545.

de la Riva, I., J. Bosch, and R. Márquez. 2001. Calls of three species of arthroleptid frogs from Río Muni, Equatorial Guinea. African Zool. 36:107–110.

de Oliveira Machado, A., G. Winck, T. A. Dorigo, and C. F. D. Rocha. 2016. Diet, diel activity pattern, habitat use, and reproductive effort of *Hylodes nasus* (Anura: Hylodidae) in one of the world's largest urban parks (Tijuca National Park), southeastern Brazil. South Am. J. Herpetol. 11:127–135.

Diaz, N. F., M. Sallaberry, and J. Valencia. 1987. Microhabitat and reproductive traits in populations of the frog, *Batrachyla taeniata*. J. Herpetol. 21:317–323.

Díaz-Páez, H., and J. C. Ortiz. 2003. Habitos alimentarios de *Pleurodema thaul* (Anura, Leptodactylidae), en Concepcion, Chile. Gayana 67:25–32.

Dodd, C. K. J. 2013a. Frogs of the United States and Canada, Vol. 1. The Johns Hopkins University Press, Baltimore, MD, USA.

Dodd, C. K. J. 2013b. Frogs of the United States and Canada, Vol. 2. The Johns Hopkins University Press, Baltimore, MD, USA.

Downie, J. R., E. G. Hancock, and A. P. Muir. 2010. The diet of the paradoxical frog *Pseudis paradoxa* in Trinidad, West Indies. Herpetol. J. 20:111–114.

Drewes, R. C. 1984. A phylogenetic analysis of the Hyperoliidae (Anura): treefrogs of Africa, Madagascar, and the Seychelles Islands. Occas. Pap. Calif. Acad. Sci. 139:1–70.

du Preez, L. and V. Carruthers. 2006. Frogs of southern Africa: A complete guide. 2nd Edition. Struik Nature, Cape Town, South Africa.

Duellman, W. E. 1961. The amphibians and reptiles of Michoacán, México. Univ. Kansas Publ. Museum Nat. Hist. 15:1–148.

Eipper, S. and P. Rowland. 2018. Frogs of Australia. John Beaufoy Publishing Ltd, Oxford, England.

Fei, L. and C. Ye. 2016. Amphibians of China. Science Press, Beijing, P.R. China.

Forlani, M. da C., P. H. Valdujo, D. Pavan, A. O. Maciel, and P. L. V. Peloso. 2011. Review of the geographical distribution of the white-spotted frog *Chiasmocleis albopunctata* (Boettger, 1885) (Anura, Microhylidae). Biota Neotrop. 11:417–421.

Formas, J. R. and M. Antonieta Vera. 1980. Reproductive patterns of *Eupsophus roseus* and *E. vittatus*. J. Herpetol. 14:11–14.

French, E. M. 2007. Response of White's Treefrog (*Litoria caerule*) to common household captivity stressors. Maryville College.

Gasc, J.-P., A. Cabela, J. Crnobrnja-Isailovic, D. Dolmen, K. Grossenbavher, P. Haffner, J. Lescure, H. Martens, J. P. Martínez Rica, H. Maurin, M. E. Oliveira, T. S. Sofianidou, M. Veith, and A. Zuidrewijk (eds). 2004. Atlas of amphibians and reptiles in Europe. Muséum national d'Histoire naturelle, Paris.

Geise, W. and K. E. Linsenmair. 1988. Adaptations of the reed frog *Hyperolius viridiflavus* (Amphibia, Anura, Hyperoliidae) to its arid environment - IV. Ecological significance of water economy with comments on thermoregulation and energy allocation. Oecologia 77:327–338.

Given, M. F. 1988. Growth rate and the cost of calling activity in male carpenter frogs, *Rana virgatipes*. Behav. Ecol. Sociobiol. 22:153–160.

Glaw, F. and M. Vences. 2007. Field guide to the amphibians and reptiles of Madagascar. Third Edit. Köln, Verlag.

Grandison, A. G. C. 1978. The occurrence of *Nectophrynoides* (Anura Bufonidae) in Ethiopia. A new concept of the genus with a description of a new species. Ital. J. Zool. 11:119–172.

Grandison, A. G. C. and S. Ashe. 1983. The distribution, behavioural ecology and breeding

strategy of the Pygmy Toad, *Mertensophryne micranotis* (Lov.). Bull. Br. Museum (Natural Hist. Zool. 45:85–93.

Griesbaum, F., M. Hirschfeld, M. F. Barej, A. Schmitz, M. Rohrmoser, M. Dahmen, F. Mühlberger, H. C. Liedtke, N. L. Gonwouo, J. Doumbia, and M.-O. Rödel. 2019. Tadpoles of three western African frog genera: *Astylosternus* Werner, 1898, *Nyctibates* Boulenger, 1904, and *Scotobleps* Boulenger, 1900 (Amphibia, Anura, Arthroleptidae). Zoosystematics Evol. 95:133–160.

Grosjean, S. 2003. A redescription of the external and buccopharyngeal morphology of the tadpole of *Ophryophryne microstoma* Boulenger, 1903 (Megophryidae). Alytes 21:45–58.

Halliday, T. 2016. The book of frogs: A life-size guide to six hundred species from around the world. Ivy Press, East Sussex, UK.

Harper, E. B., G. J. Measey, D. A. Patrick, M. Menegon, and J. R. Vonesh. 2010. Field guide to the amphibians of the eastern Arc Mountains and coastal forests of Tanzania and Kenya. Camerapix Publishers International, Nairobi.

Henderson, R. W., and R. Powell. 2009. Natural history of West Indian reptiles and amphibians. University Press of Florida, Gainesville.

Hirschmann, W., and W. Hödl. 2006. Visual signaling in *Phrynobatrachus krefftii*. Herpetologica 62:18–27.

Hoogmoed, M. S. 1977. On the presence of *Bufo nasicus* Werner in Guiana, with a redescription of the species on the basis of recently collected material. Zool. Meded. 51:265–275.

iNaturalist. 2019. <https://www.inaturalist.org/>

Inger, R. F. 1954. Systematics and zoogeography of Philippine Amphibia. Fieldiana Zool. 33:185–531.

Inger, R. F. 1966. The systematics and zoogeography of the amphibia of Borneo. Field Museum of Natural History, Chicago, USA.

Inger, R. F. 1985. Tadpoles of the forested regions of Borneo. Fieldiana Zool. 26:1–89.

Inger, R. F. and J. D. Romer. 1961. A new pelobatid frog of the genus *Megophrys* from Hong Kong. Fieldiana Zool. 39:533–538.

Irisarri, I., M. Vences, D. S. Mauro, F. Glaw, and R. Zardoya. 2011. Reversal to air-driven sound production revealed by a molecular phylogeny of tongueless frogs, family Pipidae. BMC Evol. Biol. 11:114.

IUCN. 2019. The IUCN Red List of Threatened Species. Version 2018-2.

Jones, C. 1971. Notes on hairy frogs (*Trichobatrachus robustus* Boulenger) collected in Rio Muni, West Africa. Herpetologica 27:51–54.

Kästle, W., K. Rai, and H. Schleich. 2013. Field guide to amphibians and reptiles of Nepal. ARCO-Nepal, Kathmandu.

Khan, M. S. 2006. Amphibians and reptiles of Pakistan. Krieger Publishing Company, Malabar, FL, USA.

Klages, J., F. Glaw, J. Kohler, J. Müller, C. A. Hipsley, and M. Vences. 2013. Molecular, morphological and osteological differentiation of a new species of microhylid frog of the genus *Stumpffia* from northwestern Madagascar. Zootaxa 3717:280–300.

Kok, P. J. R. and M. Kalamandeen. 2008. Introduction to the taxonomy of the amphibians of Kaieteur National Park, Guyana. Belgian Development Corporation, Brussels, Belgium.

Largen, M. and S. Spawls. 2010. The amphibians and reptiles of Ethiopia and Eritrea. Chimaira, Frankfurt am Main.

Lathrop, A., R. W. Murphy, N. L. Orlov, and C. T. Ho. 1998. Two new species of *Leptobrachium* (Anura: Megophryidae) from the central highlands of Vietnam with a redescription of *Leptobrachium chapaense*. Russ. J. Herpetol. 5:51–60.

Leenders, T. 2016. Amphibians of Costa Rica. Cornell University Press, Ithaca, NY, USA.

Liu, C.-C. 1950. Amphibians of Western China. Fieldiana Zool. Mem. 2:1–400.

Lv, Y.-Y., K. He, S. Klaus, R. M. Brown, and J.-T. Li. 2018. A comprehensive phylogeny of the genus *Kurixalus* (Rhacophoridae, Anura) sheds light on the geographical range evolution of frilled swamp treefrogs. Mol. Phylogenet. Evol. 121:224–232.

Matavelli, R., A. Martins Campos, G. R. da Silva, and G. V. de Andrade. 2014. First record of *Rhinella ocellata* (Günther, 1858) (Bufonidae) for the state of Maranhão, Northeastern Brazil. Check List J. Species List. Distrib. 10:432–433.

MacCulloch, R. D., A. Lathrop, and S. Z. Khan. 2006. Exceptional diversity of *Stefania* (Anura, Cryptobatrachidae) II: Six species from Mount Wokomung, Guyana. Phyllomedusa 5:31–41.

Malonza, P. K. 2012. Natural history observations on a Warty Frog: *Callulina dawida* (Amphibia: Brevicipitidae) in the Taita Hills, Kenya. ISRN Zool. 2012:1–9.

Martins, A. C. J. S., M. C. Kiefer, C. C. Siqueira, M. Van Sluys, V. A. Menezes, and C. F. D. Rocha. 2010. Ecology of *Ischnocnema parva* (Anura: Brachycephalidae) at the Atlantic Rainforest of Serra da Concórdia, state of Rio de Janeiro, Brazil. Zool. 27:201–208. FapUNIFESP (SciELO).

Masó, A., and M. Pijoan. 2006. Anfibios y Reptiles de la Peninsula Iberica, Baleares y Canarias. Jaguar Ediciones.

Matavelli, R., A. Martins Campos, G. R. da Silva, and G. V. de Andrade. 2014. First record of *Rhinella ocellata* (Günther, 1858) (Bufonidae) for the state of Maranhão, Northeastern Brazil. Check List J. Species List. Distrib. 10:432–433.

Meyers, P., R. Espinosa, C. S. Parr, T. Jones, G. S. Hammond, and T. A. Dewey. 2019. The Animal Diversity Web. < https://animaldiversity.org>

Mônico, A. T., T. Silva-Soares, and R. B. G. Clemente Carvalho. 2016. *Pipa carvalhoi*. Herpetol. Rev. 47:115.

Moraes, L. J. C. de L., and D. Pavan. 2018. On the occurrence of *Hemiphractus scutatus* (Spix, 1824) (Anura: Hemiphractidae) in eastern Amazonia. Amphib. Reptile Conserv. 12:5-14 (e151).

Mueses-Cisneros, J. J., D. F. Cisneros-Heredia, and R. W. McDiarmid. 2012. A new Amazonian species of *Rhaebo* (Anura: Bufonidae) with comments on *Rhaebo glaberrimus* (Günther, 1869) and *Rhaebo guttatus* (Schneider, 1799). Zootaxa 3447:22–40.

Muzzoppapa, P., and A. M. Báez. 2009. Systematic status of the mid-Tertiary neobatrachian frog *Calyptocephalella canqueli* from Patagonia (Argentina), with comments on the evolution of the genus. Ameghiniana 46:113–125.

Muñoz-Saravia, A., G. Callapa, and G. P. J. Janssens. 2018. Temperature exposure and possible thermoregulation strategies in the Titicaca water frog *Telmatobius culeus*, a fully aquatic frog of the High Andes. Endanger. Species Res. 37:91–103.

Ohler, A., S. R. Swan, and J. C. Daltry. 2002. A recent survey of the amphibian fauna of the Cardamom Mountains, Southwest Cambodia with descriptions of three new species. Raffles Bull. Zool. 50:465–481.

Pie, M. R., P. R. Ströher, R. Belmonte-Lopes, M. R. Bornschein, L. F. Ribeiro, B. C. Faircloth, and J. E. McCormack. 2017. Phylogenetic relationships of diurnal, phytotelm-breeding *Melanophryniscus* (Anura: Bufonidae) based on mitogenomic data. Gene 628:194–199.

Pikacha, P., C. Filardi, C. Morrison, and L. Leung. 2016. Factors affecting frog density in the

Solomon Islands. Pacific Conserv. Biol. 22:223–235.

Plath, M., M. Solbach, and H.-W. Herrmann. 2004. Anuran habitat selection and temporal partitioning in a montane and submontane rainforest in Southwestern Cameroon – first results. Salamandra 40:239–260.

Pombal, J. P., I. Sazima, and C. F. B. Haddad. 1994. Breeding behavior of the Pumpkin Toadlet, *Brachycephalus ephippium* (Brachycephalidae). J. Herpetol. 28:516–519.

Portik, D. M., and D. C. Blackburn. 2017. The evolution of reproductive diversity in Afrobatrachia: A phylogenetic comparative analysis of an extensive radiation of African frogs. Evolution 70:2017–2032.

Portik, D. M., G. F. M. Jongsma, M. T. Kouete, L. A. Scheinberg, B. Freiermuth, W. P. Tapondjou, and D. C. Blackburn. 2018. Ecological, morphological, and reproductive aspects of a diverse assemblage of hyperoliid frogs (Family: Hyperoliidae) surrounding Mt. Kupe, Cameroon. Herpetol. Rev. 49:397–408.

Poyarkov Jr., N. A., A. B. Vassilieva, N. L. Orlov, E. A. Galoyan, T. T. A. Dao, D. T. T. Le, V. D. Kretova, and P. Geissler. 2014. Taxonomy and distribution of narrow-mouth frogs of the genus *Microhyla* Tschudi, 1838 (Anura: Microhylidae) from Vietnam with descriptions of five new species. Russ. J. Herpetol. 21:89–149.

Rabb, G. B., and M. S. Rabb. 1963. On the behavior and breeding biology of the African pipid frog: *Hymenochirus boettgeri*. Z. Tierpsychol. 20:215–241.

Rabearivony, J., A. P. Raselimanana, M. A. Andriamazava, R. Thorstrom, and L. A. R. de Roland. 2010. A new locality for the endangered microhylid frog *Scaphiophryne boribory* from northern Madagascar and a rapid survey of other amphibians of the Bemanevika region. Herpetol. Notes 3:105–109.

Richards, S. J. 2007. A new species of *Nyctimystes* (Anura: Hylidae) from Papua New Guinea. Phyllomedusa 6:105–118.

Rödel, M.-O. 2000. Herpetofauna of West Africa. Vol 1. Amphibians of the West African savanna. Chimaira, Frankfurt am Main.

Rowley, J. J. L., D. T. T. Le, D. T. A. Tran, and H. D. Hoang. 2011. A new species of *Leptolalax* (Anura: Megophryidae) from southern Vietnam. Zootaxa 2796:15–28.

Sanderson, I. T. 1936. The amphibians of the Mamfe Division, Cameroons – II. Ecology of the frogs. J. Zool. 106:165–208.

Savage, J. M. 1972. The harlequin frogs, genus *Atelopus*, of Costa Rica and Western Panama.

Herpetologica 28:77–94.

Savage, J. M. 2002. The amphibians and reptiles of Costa Rica. A herpetofauna between two continents, between two seas. University of Chicago Press, Chicago.

Schleich, H. H. and W. Kästle (eds). 2002. Amphibians and reptiles of Nepal. Gatner Verlag, Koenigstein, Germany.

Segev, O., F. Andreone, R. Pala, G. Tessa, and M. Vences. 2012. Reproductive phenology of the tomato frog, *Dyscophus antongili*, in an urban pond of Madagascar's east coast. Acta Herpetol. 7:331–340.

Senevirathne, G., S. Garg, R. Kerney, M. Meegaskumbura, and S. D. Biju. 2016. Unearthing the fossorial tadpoles of the Indian dancing frog family Micrixalidae. PLoS One 11:1–18.

Singer, R. S. and M. Gutiérrez. 1997. Reproducción y desarrollo larval del sapo enano *Melanophryniscus stelzneri stelzneri* (Weyemberg, 1875) (Anura: Bufonidae). Cuad. Herpetol. 11:21–30.

Staniszewski, M. 1998. Madagascan Burrowing Frogs FAQ. <http://www.amphibian.co.uk/scaphiophryne.html>

Stuart, B. 2005. New frog records from Laos. Herpetol. Rev. 36:473–479.

Stuart, B. L., K. Sok, and T. Neang. 2006. A collection of amphibians and reptiles from hilly eastern Cambodia. Raffles Bull. Zool. 54:129–155.

Tinsley, R. C. 1990. The influence of parasite infection on mating success in spadefoot toads, *Scaphiopus couchii*. Am. Zool. 30:313–324.

Vásquez, T., and K. S. Pfennig. 2007. Looking on the bright side: Females prefer coloration indicative of male size and condition in the sexually dichromatic spadefoot toad, *Scaphiopus couchii*. Behav. Ecol. Sociobiol. 62:127–135.

Wells, E., D. Garcia-Alonso, G. M. Rosa, and B. Tapley. 2015. Amphibian Taxon Advisory Group Best Practice Guidelines for Midwife toads (*Alytes* sp.).

Zug, G. R. 2015. Morphology and systematics of *Kalophrynus interlineatus-pleurostigma* populations (Anura: Microhylidae: Kalophryninae) and a taxonomy of the genus *Kalophrynus* Tschudi, Asian Sticky Frogs. Proc. Calif. Acad. Sci. 62:135–190.

Zweifel, R. G. 1979. A new cryptic species of microhylid frog (genus *Cophixalus*) from Papua New Guinea, with notes on related forms. Am. Museum Novit. 1–14.

Personal observations used in assigning each anuran species to ecological traits/states (Table S1)

AC = Alan Channing (University of the Western Cape)
BLS = Bryan L. Stuart (North Carolina Museum of Natural Sciences)
DJG = David Gower (Natural History Museum)
DV = Deepak Veerappan (Natural History Museum)
FK = Fred Kraus (University of Michigan)
JL = Jim Labisko (University of Kent)
JWS = Jeff Streicher (Natural History Museum)
MOR = Mark-Oliver Rödel (Museum für Naturkunde, Berlin)
RCB = Rayna C. Bell (Smithsonian National Museum of Natural History)
RDS = Rafael de Sá (University of Richmond)
SM = Stephen Mahoney (Natural History Museum)
SPL = Simon Loader (Natural History Museum)
YC = Yodchaiy Chuaynkern (Khon Kaen University)
